# Age and Sex Differences in Carotid Intima-Media Thickness: A Systematic Review and Meta-Analysis

**DOI:** 10.3390/life14121557

**Published:** 2024-11-27

**Authors:** Veronika A. Myasoedova, Alessio L. Ravani, Beatrice Frigerio, Donato Moschetta, Vincenza Valerio, Ilaria Massaiu, Mauro Amato, Damiano Baldassarre, Paolo Poggio

**Affiliations:** 1 Centro Cardiologico Monzino IRCCS, 20138 Milan, Italy; veronika.myasoedova@ccfm.it (V.A.M.); alessio.ravani@ccfm.it (A.L.R.); beatrice.frigerio@ccfm.it (B.F.); donato.moschetta@ccfm.it (D.M.); vincenza.valerio@ccfm.it (V.V.); ilaria.massaiu@ccfm.it (I.M.); mauro.amato@ccfm.it (M.A.); damiano.baldassarre@ccfm.it (D.B.); 2 Department of Medical Biotechnology and Translational Medicine, University of Milan, 20129 Milan, Italy; 3 Department of Biomedical, Surgical and Dental Sciences, University of Milan, 20122 Milan, Italy

**Keywords:** carotid artery intima-media thickness, sex difference, carotid atherosclerosis, ageing

## Abstract

Background: Ageing is a significant risk factor for carotid atherosclerosis, affecting over a billion people worldwide. Carotid intima-media thickness (cIMT) is a surrogate marker for cardiovascular disease (CVD) risk, with age- and sex-related differences in levels and progression. The onset of clinical manifestations of CVD in women is delayed by about 10 years compared to men. The present study aims to evaluate whether subclinical atherosclerosis is the same disease in men and women or two pathologies with a possible different etiology. For this purpose, we analyzed the differences in cIMT, the impact of patient characteristics, and the influence of age on cIMT in men and women. Methods: A systematic search related to cIMT measured by an ultrasound and gender-specific differences was conducted according to the PRISMA 2020 guidelines. Ninety studies, enrolling 165,551 subjects (76,955 men and 88,553 women), were included in the quantitative synthesis. Results: We found that men compared to women had greater common cIMT, (standardized mean difference (SMD) = 0.506, *p* < 0.03; I^2^: 98.2, *p* < 0.0001), greater bifurcation IMT (SMD = 1.056, *p* = 0.022; I^2^: 99.9%, *p* < 0.001), and higher internal cIMT (SMD = 1.124, *p* = 0.017; I^2^: 99.9%, *p* < 0.001). The study did not reveal any association between cardiovascular risk factors and differences in cIMT between men and women. A virtual analysis revealed that age-related cIMT is equal between sexes but postponed by 10 years in women. Conclusions: This study showed that classical risk factors for CVD have a comparable effect on cIMT in men and women. While subclinical atherosclerotic disease, as assessed by cIMT, is essentially identical in the two sexes, it manifests itself by about 10 years later in women.

## 1. Introduction

The prominent role of ageing as a risk factor for carotid atherosclerosis indicates that a large number of people affected by atherosclerosis are expected in the context of global demographic ageing [1]. A recent meta-analysis of 59 studies shows that approximately 28% of individuals in the general population had increased carotid intima-media thickness (cIMT), translating to just over one billion people worldwide [1]. Carotid IMT can be considered a surrogate marker of cardiovascular disease (CVD) risk, given the significant association between the positive effects of treatment on cIMT progression and decreased risk of CVD demonstrated in a meta-analysis of 119 randomized clinical trials and more than 100,000 participants [2]. It is also known that there are age- and sex-related differences in cIMT levels and progression [3,4] as well as sex-specific risk factors [5]. In particular, smoking and alcohol consumption were significantly associated with greater cIMT changes in women, whereas hypertension was associated with increased cIMT progression in men [5,6].

The gap in the development and progression of CVD between sexes is approximately 10 years and being delayed in women [7]. Numerous features, such as genetic, inflammatory, and immune factors, as well as endocrine components, are responsible for this phenomenon [7]. Thus, we need to better understand the mechanisms of vascular ageing and develop/implement novel age- and sex-related targeted-preventive strategies.

The aims of the present systematic review and meta-analysis were: (1) to evaluate whether subclinical atherosclerosis is the same disease in men and women using a cIMT assessment in the largest known combined analysis; (2) to assess the impact of patients’ clinical characteristics on cIMT alterations between men and women; (3) to estimate the impact of age on cIMT in men and women.

## 2. Methods

### 2.1. Data Sources and Searches

A detailed protocol for the search strategy of this review was developed prospectively, specifying objectives, study selection criteria, outcomes, and statistical methods.

To identify all available studies on the association between sex and cIMT in the general population and in subjects with CVD, a systematic search of electronic databases (PubMed, Web of Science, and Scopus) according to the PRISMA 2020 guidelines [8] was performed. The search string applied to PubMed was the following: (carotid intima media thickness OR IMT OR carotid intima-media thickness) AND (gender difference OR sex difference). The last search was performed in June 2022.

### 2.2. Study Selection, Data Extraction, and Quality Assessment

According to the pre-established protocol, all studies that evaluated cIMT, the measurement of the thickness of the innermost two layers of the arterial wall (a widely-used marker for atherosclerosis and cardiovascular risk assessment), in men and women and the impact on cardiovascular risk factors were included. Case reports, reviews, and articles on animal models were excluded. To be included in the analysis, a study had to provide values (means with standard deviation) of cIMT, in men and women, separately, measured in millimeters. If the median and 95% confidence interval (CI) were present, the data were transformed into the mean and standard deviation [9]. We included the studies where common carotid artery (CCA), carotid bifurcation (Bif), and internal carotid artery (ICA) IMT were measured by the B-mode ultrasound. Two types of analyses were performed: (1) difference between men and women in cIMT; (2) age-related changes in cIMT in men and women.

### 2.3. Statistical Analysis and Risk of Bias Assessment

A statistical analysis was performed using Comprehensive Meta-analysis Version 3.3.070 (Biostat, Englewood, NJ, USA, 2014). The differences in continuous variables were expressed as the standardized mean difference (SMD) and 95% CI. The overall effect was tested using Z scores and significance was set at *p* < 0.05. The statistical heterogeneity among studies was assessed with the chi-square Cochran Q test and with the I^2^ statistic, which measures the inconsistency across study results and describes the proportion of total variation in study estimates that is due to heterogeneity rather than the sampling error. In detail, I^2^ values of 0% indicate no heterogeneity, <25% low, 25–50% moderate, and 50% or more high heterogeneity [10]. Pearson’s correlation coefficient was used to measure the linear association between cIMT and age in both sexes.

The evaluation of the methodological quality of each study was performed according to the Newcastle–Ottawa Scale (NOS). The scoring system encompasses three major domains (selection, exposure, and outcome) with a resulting score ranging between 0 and 9, a higher score representing a better methodological quality. Two authors (RA and VAM) analyzed each article and performed the data extraction, separately and in the case of disagreement, a third investigator was consulted (PP). Discrepancies were resolved by consensus. Egger’s test and funnel plots of the logit event rate vs. the standard error were used as a graphical representation to evaluate the risk of bias. Publication bias was assessed by a visual inspection of funnel plots and Egger’s test with *p* < 0.05 being considered as statistically significant [11]. The random-effect method was used for all analyses to consider the variability among the included studies. In the case of a significant publication bias, Duval and Tweedie’s trim and fill method was used to allow for the estimation of the adjusted effect size [12].

### 2.4. Meta-Regression Analysis

The differences between men and women in cIMT may be affected by differences in the clinical and demographic characteristics of patients included in different studies [mean age, body mass index (BMI), total cholesterol (TC), low density lipoprotein (LDL), high density lipoprotein (HDL), triglycerides (TGs)] as well as the presence of cardiovascular risk factors (i.e., hypertension, diabetes, dyslipidemia, and smoking), and the presence of coronary artery disease (CAD). To assess the possible effect of such variables in explaining the different results observed across studies, the meta-regression analysis after implementing a regression model with the cIMT standardized mean difference as dependent variables (y) and the variables mentioned above as independent variables (x) was performed [13]. In the case of alternative decisions or ranges of values for decisions that were arbitrary or unclear, a sensitivity analysis was performed.

## 3. Results

**Figure 1:**
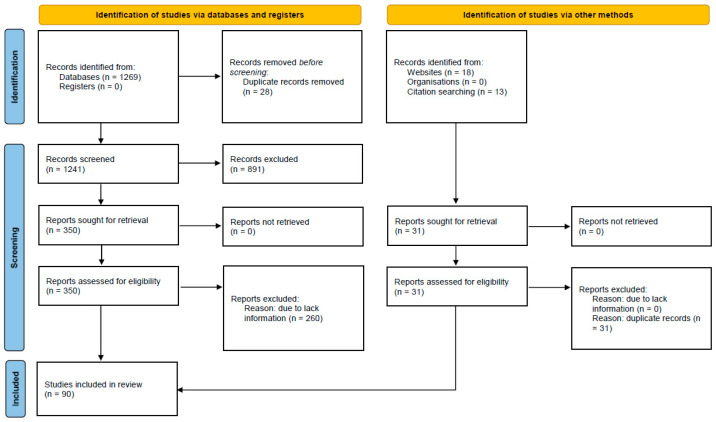
Prisma Flow Diagram. The flow chart represents the number of studies evaluated according to the PRISMA 2020 guidelines.

The literature search retrieved 1269 studies and duplicate results were excluded. After a screening of titles and abstracts, 350 articles were selected for full-text evaluation. The revision of full-length papers allowed for the exclusion of 260 studies due to a wrong study design or irrelevant information in their content. A total of 90 studies, enrolling 165,551 subjects (76,955 men and 88,553 women), were included in this systematic review and meta-analysis (Figure 1, Table 1).

### 3.1. Study Characteristic

Seventy studies on general population subjects and 20 studies on patients with CVD provided information on CCA IMT (Table 1). Of all the studies reviewed, 11 reported information also on the mean IMT of carotid Bif, whereas 14 provided data on ICA wall parameters. The mean cIMT was 0.682 mm (range: 0.370–2.111 mm) in women and 0.725 mm (range: 0.400–1.390 mm) in men. The overall mean age was 51 years (range: 15–73 years), mean BMI 26.8 kg/m^2^ (range: 21.8–41.4 kg/m^2^), mean TC 199 mg/dL (range: 159–256 mg/dL), mean LDL 127 mg/dL (range: 83–186 mg/dL), mean HDL 52 mg/dL (range: 41–69 mg/dL), and mean TG 119 mg/dL (range: 74–168 mg/dL). Concerning the cardiovascular risk factors, hypertension was present in 44% of participants (range: 21–80%), diabetes in 21% (range: 1–100%), dyslipidemia in 40% (range: 3–84%), and smoking in 25% (range: 9–91%). CAD was present in 7% of participants (range: 0.3–30%). Clinical and demographic characteristics of the included cohorts are summarized in Supplementary Tables S1–S3.

### 3.2. Common Carotid Artery Intima-Media Thickness in Men and Women

**Figure 2:**
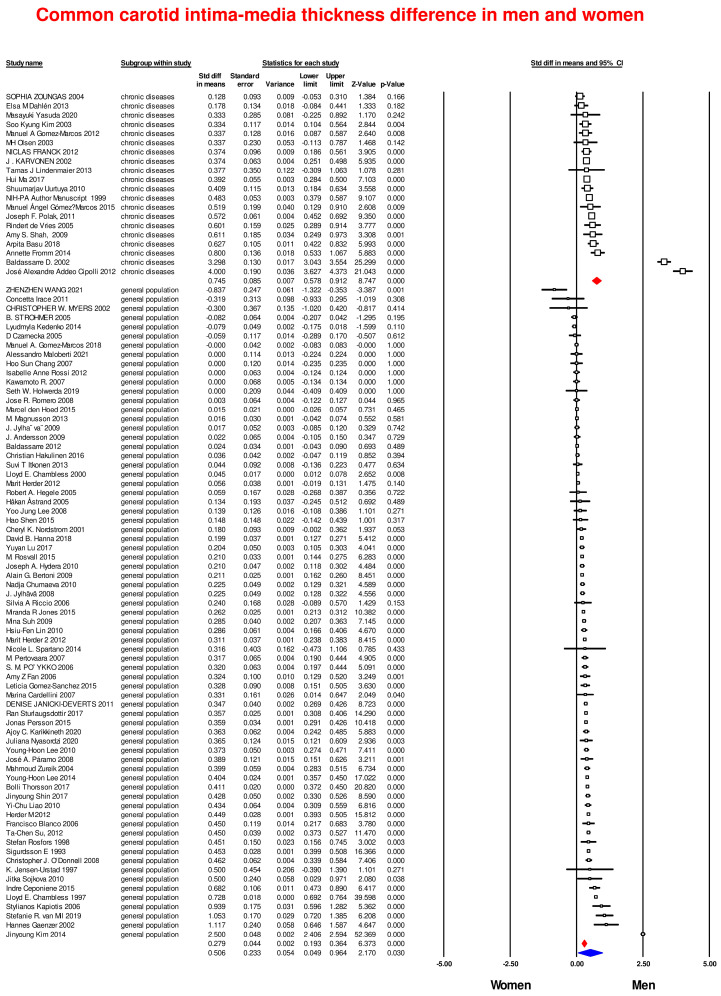
Forest plots of carotid intima-media thickness differences in men and women in general population subjects and in patients with cardiovascular disease. Differences in common carotid artery intima-media thickness (CCA IMT) were evaluated with the standardized difference in means (SMD) between men and women. The diamonds represent the estimated overall effect, whereas the squares represent each study with 95% CI.

The evaluation of CCA IMT showed that men had a significant increased thickness compared to women with a SMD of 0.506 (CI 95%: 0.49–0.96, *p* = 0.03; Figure 2) and a high heterogeneity among studies (I^2^: 98.2; *p* < 0.0001). Similar results were obtained analyzing separately general population studies and studies including patients with CVD. The difference in CCA IMT remained significant in both groups with greater values in men compared to women (SMD = 0.279, 95% CI: 0.19–1.36, *p* < 0.001 and SMD = 0.745, CI 95%: 0.58–0.91, *p* < 0.001, respectively; Figure 2).

### 3.3. Bifurcation and Internal Carotid Artery Intima-Media Thickness (IMT) in Men and Women

**Figure 3:**
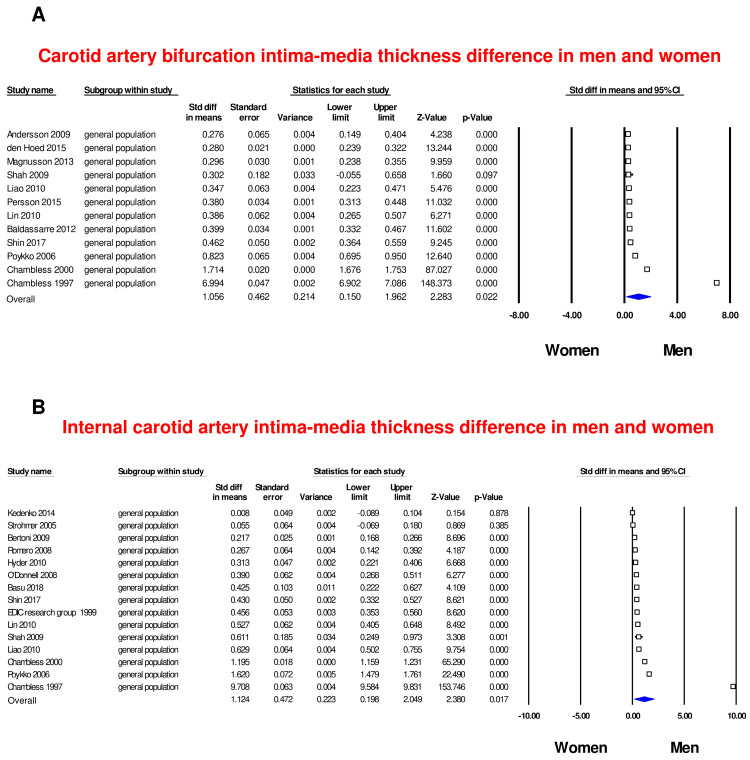
Forest plots of carotid intima-media thickness differences in men and women. Differences in bifurcation (Bif) (**A**) and internal carotid artery (ICA) intima-media thickness (IMT) (**B**) were evaluated with the standardized difference in means (SMD) between men and women. The blue diamond represents the estimated overall effect, while the squares represent each study with 95% CI.

Often, the decision to measure cIMT solely in the CCA is justified by the ease and accuracy of measurements in this segment, in comparison to the Bif or ICA. Nonetheless, cIMT of the Bif and ICA is more prone to be affected by flow turbulence than that of the CCA [14]. Thus, to get a broader view of the differences between men and women in IMT, we performed a sub-analysis by including additional carotid segments, such as the carotid Bif with 12 studies (Supplementary Table S2) and the ICA with 15 studies (Supplementary Table S3). For this analysis, only general population cohorts were considered, because of the lack of data from studies on CVD patients. We found that in both Bif and ICA segments, men had greater mean IMT compared to women (SMD = 1.056, 95% CI: 0.15–1.96, *p* = 0.022 and SMD = 1.124, 95% CI: 0.20–2.05, *p* = 0.017, respectively; Figure 3A,B). Of note, the SMDs of these segments were at least doubled compared to the CCA IMT. The heterogeneity among the studies was significant in both groups (I^2^: 99.9%, *p* <  0.001).

### 3.4. Meta-Regression Analysis

Meta-regression models, applied for total numbers of studied cohorts, did not reveal any association among the cardiovascular risk factors considered and the difference in CCA IMT between men and women (Supplementary Table S4). Analyzing patients with and without CVD separately, we identified that only in general population studies, a positive association was observed between the triglyceride level and difference in CCA IMT between men and women (Z-value: 2.53; *p* = 0.012, Supplementary Table S5), whereas in the population with CVD, no significant correlations between the cardiovascular risk factors and difference in CCA IMT in men and women were observed 0.001, Supplementary Table S6.

We performed further meta-regression analysis of studies including the measurement of Bif and ICA. In such analysis, we did not observe any significant effect modifiers in the association between cIMT differences in men and women, for both studied groups (Supplementary Tables S7 and S8).

### 3.5. Age-Related Differences Between Men and Women in Carotid Intima-Media Thickness (cIMT)

Since age is one of the most important confounding factors associated with cIMT, we performed a further analysis dividing the population studied in two groups: younger and older than 50 years old (Supplementary Figure S1). In both patients older than 50 years and those younger than 50 years, males had higher CCA IMT with a 1.4-fold increase in the former group (SMD = 0.422, CI 95%: 0.325–0.519, *p* < 0.001 and SMD = 0.294, CI 95%: 0.163–0.424, *p* < 0.001, respectively).

**Figure 4:**
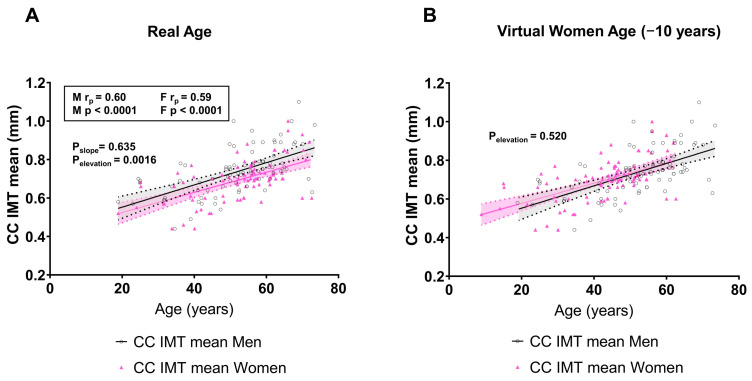
Correlation between carotid intima-media thickness and age in men and women. (**A**) The correlation plot indicates the results of 90 studies with real age in men and women and mean common carotid artery (CCA) intima-media thickness (IMT) levels. The abscissa (x axis) represents the mean age, whereas the ordinate (y axis) represents the mean CCA IMT in each included study. (**B**) The correlation plot indicates the same studies but using a virtual age of women (−10 years) compared with the real age of men and mean CCA IMT levels.

Epidemiological, clinical, and experimental data have shown that there is a cardiovascular time gap, averaging about 10 years, between the two sexes [7]. Analyzing the total number of cohorts by age decades (younger than 20 years, 21–40, 41–60, 61–80), we found that men had higher cIMT levels at all time points compared to women (regression lines P_elevation_ = 0.0016), but the slopes were almost identical (P_slope_ = 0.635), indicating the absence of a significant difference between men and women in mean cIMT age-related changes (Figure 4A). In addition, considering the 10-year gender-specific gap, we virtually corrected the cIMT levels, by shifting the cIMT data of women towards one decade to the left (Figure 4B). Of note, this analysis highlights that the previous difference was completely abrogated (P_elevation_ = 0.520), corroborating the hypothesis that over time, the change in women cIMT is similar to that of men but with a 10-year gap shift.

### 3.6. Publication Bias

Funnel plots of effect size versus standard error for all the performed analyses were rather symmetrical. Egger’s test showed the absence of publication bias for studies including the IMT of CCA, general population (*p* = 0.812; Supplemental Figure S2A) and CVD groups (*p* = 0.221; Supplemental Figure S2B), for studies including the IMT of Bif (*p* = 0.881; Supplemental Figure S3A) and studies including the IMT of ICA (*p* = 0.752; Supplemental Figure S3B). All studies included in this analysis had the NOS for quality ≥ 5; and the median value quality assessment was six (Supplementary Table S9). Thus, a sensitivity analysis was not performed according to the quality of the studies.

## 4. Discussion

The tesults of our meta-analysis, performed on 90 studies with more than 150,000 subjects, consistently showed that, regardless of the carotid artery segment considered or the type of population (general or patients with CVD), men had greater mean cIMT compared to women independently of age. The meta-regression analysis did not reveal any association between the cIMT sex difference and classical CVD risk factors. Accordingly, we infer that these factors influence at the same extent cIMT in men and women. Finally, similarly to the demonstrated sex-specific gap in vascular events, we highlighted for the first time that, also considering a marker of subclinical atherosclerosis (i.e., cIMT), the relationship between cIMT and age is substantially identical in the two sexes but postponed by about 10 years in women.

The high correlation of cIMT measured by the B-mode ultrasound with a vessel wall thickness measured in histological specimens [15], the association with risk factors and prevalence of CVD [16], the high correlation with atherosclerosis in other vessels [17], the association with the occurrence of clinical events [18,19,20], and the possibility of influencing cIMT with therapeutic interventions [21], lead us to consider cIMT as an essential modifiable player in CVD onset and prevention [14]. Indeed, recent evidence shows a compatible effect of the intervention on CCA IMT progression and CVD risk, suggesting the usefulness of cIMT progression as a surrogate marker in clinical trials, particularly in studies dedicated to future cardiovascular drug development [2]. The total majority of the atherosclerosis studies use CCA IMT, the distal 1 cm, a segment unlikely affected by atherosclerosis [22]. Whereas the bifurcation and ICA better reflect clinically relevant cIMT progression [23]. Indeed, the results of our meta-analysis of little numbers of studies that included the measurement of these two segments indicated a double SMD between men and women compared to the CCA IMT.

Carotid IMT exhibits the ability to change continuously with advancing age [4,24]. Furthermore, the presence of CVD and/or CV risk does not affect this consistent increase in cIMT over the life span [25]. Male sex is often associated with an early onset and progression of CVD compared to female sex; indeed, age, sex, and age–sex interaction have been shown to be strong predictors of cIMT progression [26,27,28]. In agreement with several previous reports, we observed significantly higher levels of cIMT in men compared to women, over time. Taking into account that measuring cIMT in the whole carotid tree may provide a more consistent association [14], we performed our analysis on CCA, Bif, and ICA; however, in all examined segments, men showed higher mean cIMT levels than women.

The protective effect of female sex hormones has a better impact on risk factors in women, and it has been reported that cIMT is greater in women with early surgical menopause [29], indicating that a shorter exposure to female hormones may influence the progression of atherosclerosis. The positive effect of estrogens on metabolism, macrophages, and smooth muscle cell function, slows down atherosclerosis progression [30]. However, dividing our study cohort into groups of age less than or greater than 50 years, the mean age of menopause in women, men consistently showed higher levels of cIMT than women. In addition, our results show that cIMT growth curves of men and women increased in parallel, indicating no significant differences between men and women in age-related mean cIMT changes. Indeed, when we adjusted the analysis for the known cardiovascular gap, the regression lines between men and women were superimposable, corroborating the positive effects of estrogen.

Other than age, several factors, such as smoking, obesity, diabetes, dyslipidemia, and hypertension have been shown to be associated with a one-time measured increase in cIMT levels and changes over time in both sexes [31]. However, the results of our meta-regression analysis, which assessed the sex-specific association of cIMT with lipids, BMI, the presence of cardiovascular risk factors (i.e., hypertension, diabetes, dyslipidemia, and smoking), and the presence of CAD, did not reveal any influential risk factors that could explain the difference between men and women in cIMT levels, suggesting that classical risk factors for CVD strongly influence cIMT with an equivalent strength in men and women.

Recent evidence also points to sex-specific genetic and systemic inflammatory effects on cIMT [32,33,34]. Indeed, gene activity is highly influenced by sex and varies across multiple tissues [35,36] and sex steroids may interfere with gene expression through various mechanisms, such as an interaction with epigenetic modifiers [35], possibly leading to IMT sex-specific differences. Additionally, sex differences in systemic inflammatory profiles may influence the atherosclerosis processes in a sex-dependent manner [37]. Indeed, men have been reported to have more circulating CD14^+^ and CD16^+^ monocytes, which are associated with impaired endothelial function, increased IMT, and reduced carotid compliance compared to women [38,39,40,41].

A separate analysis on the general population and patients with CVD highlighted the association between TG and TC with the difference between men and women in mean cIMT, respectively. In a recent study of patients at high CV risk, it was reported that the association between dyslipidemia and progression of carotid atherosclerosis was stronger in men, suggesting that TC is a better predictor of atherosclerosis in women, whereas TG, in line with our results, showed better prediction in men [5]. However, there are few studies dedicated to assessing sex-specific lipid profiles and cIMT levels and changes and those that are available provide contradictory results [24,42]. Thus, future dedicated studies are needed to evaluate the sex-specific influence of CV risk factors, with a particular emphasis on lipid profile and subclinical atherosclerosis.

### 4.1. Clinical Implication

Based on our results, we can safely assume that preventive strategies aiming at reducing atherosclerosis and CVD risk should be similar in men and women. However, the temporary gap between sexes should be taken into account when screening procedures are performed. Indeed, these actions should be carried out with a different starting point according to sex, in men at least 10 years earlier than in women. In addition, the age gap in sex-specific cIMT levels could be applied in pharmacological trials to optimize the evaluation of drug efficacy. Finally, not only CCA IMT, but also Bif and ICA, should be evaluated to assess the effect of the therapy and disease progression.

### 4.2. Study Limitations

Our study has potential limitations. First, our systematic review and meta-analysis were not prospectively registered as suggested by the Cochrane guidelines. Nevertheless, we followed the latest PRISMA guidelines (2020). Second, we performed our analysis of carotid artery mean IMT, without consideration of the maximum IMT or carotid plaque. We did not consider plaques for the following reasons: (i) the criteria utilized for the definition of carotid plaque are not well established, (ii) several strategies of plaque measurement were applied in the included studies, and iii) in most of the paper, plaques were categorized only in terms of presence/absence. Third, racial aspects of cIMT were not considered in this study, which may affect the generalizability of our findings to different racial groups. Finally, the evaluation of IMT difference in men and women in carotid Bif and ICA was performed including only general population cohorts, due to the insufficient numbers of studies aimed to evaluate the IMT of Bif and ICA in patients with CVD.

## 5. Conclusions

In conclusion, this meta-analysis, besides corroborating the evidence that men have a higher mean cIMT than women, regardless of age or carotid artery segment measured, shows that classical risk factors for CVD have a comparable effect on cIMT in both sexes, leading to the same subclinical atherosclerotic disorder, but postponed by 10 years in women. Overall, the cIMT measurement is a valuable tool in assessing CVD risk and its modification through therapeutic interventions may represent an effective strategy in CVD prevention. However, to account for the observed sex differences in cIMT, a theoretical gap of 10 years should be considered.

## Figures and Tables

**Table 1:** Studies included in the meta-analysis on the difference in common carotid intima-media thickness between men and women.

Author + Year	Men, n	CCA IMT Mean	Women, n	CCA IMT Mean
Jensen-Urstad 1997	10	0.59	10	0.55
Nordstrom 2001	249	0.69	218	0.67
Myers 2002	15	0.50	15	0.53
Olsen 2003	73	1.00	26	0.95
Kim 2003	240	0.75	106	0.70
Zoungas 2004	355	0.79	173	0.77
Zureik 2004	583	0.72	579	0.69
Czarnecka 2005	135	0.66	159	0.68
Hegele 2005	59	0.78	91	0.77
Riccio 2006	118	0.88	51	0.84
Blanco 2006	132	0.98	160	0.89
Polak 2011	584	0.64	532	0.59
Herder 2012_1	1307	0.73	1436	0.69
Lindenmaier 2013	24	0.78	15	0.73
Fromm 2014	122	0.88	112	0.72
Gomez-Sanchez 2015	272	0.75	228	0.72
Lu 2017	711	0.63	888	0.60
van Mil 2019	52	0.64	148	0.53
Chambless 1997	5552	0.66	7289	0.60
Chambless 2000	6349	0.66	7865	0.60
Hoed 2015	4055	0.76	5076	0.71
Magnusson 2013	1915	0.74	2821	0.70
Baldassarre 2012	1666	0.74	1780	0.70
Andersson 2009	474	0.88	475	0.85
EDIC group 1999	762	0.69	705	0.66
Baldassarre 2002	245	0.77	314	0.70
Karvonen 2002	505	1.00	519	0.84
Gaenzer 2002	76	0.58	26	0.44
Strohmer 2005	851	0.75	348	0.76
Astrand 2005	50	0.60	58	0.58
de Vries 2005	98	0.89	71	0.79
Poykko 2006	509	0.87	515	0.76
Cardellini 2007	58	0.77	118	0.73
Fan 2006	231	0.71	182	0.68
Kawamoto 2007	388	0.93	480	0.93
Pertovaara 2007	439	0.60	541	0.57
Chang 2007	130	0.68	150	0.68
Lee 2008	113	0.69	143	0.68
O’Donnell 2008	496	0.64	566	0.58
Romero 2008	439	0.70	567	0.67
Bertoni 2009	3089	0.89	3393	0.85
Jylhävä 2008	704	0.59	994	0.57
Páramo 2008	311	0.73	89	0.66
Suh 2009	1054	0.95	1595	0.88
Jylha 2009	891	0.59	618	0.58
Shah 2009	50	0.58	79	0.52
Hydera 2010	929	0.89	904	0.85
Sojkova 2010	42	0.70	31	0.60
Lee 2010	634	0.75	1096	0.70
Uurtuya 2010	142	0.89	170	0.73
Liao 2010	482	0.64	531	0.59
Chumaeva 2010	719	0.59	1002	0.57
Lin 2010	496	0.63	587	0.59
Irace 2011	33	0.68	15	0.72
Franck 2012	309	0.76	173	0.69
Rossi 2012	465	0.70	546	0.70
Janicki-Deverts 2011	1283	0.70	1276	0.66
Gomez-Marcos 2012	153	0.73	105	0.69
Herder 2 2012	1442	0.84	1532	0.79
Cipolli 2012	131	0.79	207	0.75
Su 2012	1203	0.74	1487	0.66
Dahlén 2013	172	0.70	83	0.67
Itkonen 2013	176	0.57	370	0.54
Kim 2014	1624	0.71	1505	0.70
Spartano 2014	12	0.49	13	0.46
Kedenko 2014	1107	0.76	663	0.77
Lee 2014	3019	0.82	4535	0.77
Rosvall 2015	1522	0.77	2212	0.74
Jones 2015	2991	0.90	3356	0.85
Persson 2015	1653	0.74	1777	0.70
Ceponiene 2015	168	0.66	212	0.61
Gómez-Marcos 2015	71	0.78	41	0.72
Shen 2015	95	0.54	88	0.52
Hakulinen 2016	1018	0.59	1247	0.57
Shin 2017	723	0.57	956	0.52
Thorsson 2017	4843	0.78	5700	0.73
Sigurdsson 1993	2629	0.77	2719	0.72
Herder 2012_2	2214	0.79	2981	0.73
Yasuda 2020	56	1.10	16	1.00
Holwerda 2019	52	0.44	41	0.44
Gómez-Marcos 2018	1456	0.73	898	0.73
Hanna 2018	1304	0.73	1722	0.71
Basu 2018	269	0.68	148	0.61
Sturlaugsdottir 2017	3204	0.76	3320	0.71
Ma 2017	512	0.75	958	0.70
Rosfors 1998	90	0.89	92	0.80
Kapiotis 2006	75	0.40	70	0.37
Nyasordzi 2020	120	0.57	145	0.55
Maloberti 2021	273	0.60	106	0.60
Karikkineth 2020	512	0.76	555	0.70
Wang 2021	32	0.551	40	0.599

CCA IMT: common carotid artery intima-media thickness; n: number.

## Data Availability

The manuscript entirely reports published data. Spreadsheets with data as compiled for the present manuscript can be made available on reasonable request to the corresponding author.

## Supplementary Materials

The following supporting information can be downloaded at: https://www.mdpi.com/article/10.3390/life14121557/s1, Table S1: Clinical and demographic characteristics of subjects included in studies where intima-media thicknesses of the common carotid artery were measured; Table S2: Clinical and demographic characteristics of subjects included in studies where intima-media thicknesses of carotid artery bifurcation were measured; Table S3: Clinical and demographic characteristics of subjects included in studies where intima-media thicknesses of the internal carotid artery were measured; Table S4: Meta-regressions analysis for studies included measurements of intima-media thickness in common carotid artery; Table S5: Meta-regressions analysis for general population studies. Table S6: Meta-regressions analysis for cardiovascular disease studies; Table S7: Meta-regressions analysis for studies included intima-media thickness measurement of carotid bifurcation; Table S8: Meta-regressions analysis for studies included intima-media thickness measurement of internal carotid artery; Table S9: Quality assessment with Newcastle-Ottawa scale of the included studies; Figure S1: Forest plots of common carotid artery intima-media thickness (CCA IMT) difference in men and women in subjects younger and older than 50 years old. Difference in IMT was evaluated with the standardized difference in means (SMD) between men and women. The diamond represents the estimated overall effect, while the squares represent each study with 95% CI; Figure S2: Funnel plots of effect size versus standard error for studies measured intima-media thickness of common carotid artery. (A) Studies included general population cohorts. (B) Studies included cardiovascular disease patients; Figure S3: Funnel plots of effect size versus standard error for studies measured intima-media thickness of carotid artery bifurcation (A) and internal carotid artery (B).
