# Supplementary material for: Age and Sex Differences in Carotid Intima-Media Thickness: A Systematic Review and Meta-Analysis
Source: Life (Basel). 2024 Nov 27;14(12):1557. doi: 10.3390/life14121557 (PMC11678405; doi:10.3390/life14121557)
Supplement: Supplementary file 1 [file life-14-01557-s001.zip › life-3217667-supplementary.pdf]

SUPPLEMENTARY TABLES AND FIGURES

Supplementary Table S1. Clinical and demographic characteristics of subjects included in studies where intima-media thicknesses of the common carotid artery were measured.

Author + Year	Study type	Population	Men, n	Age	CCA IMT mean	Women, n	Age	CCA IMT mean	BMI, kg/m2	TC, mg/dL	LDL, mg/dL	HDL, mg/dL	TG, mg/dL	HT, %	DM, %	DLD, %	CAD, %	Smoking, %
Jensen-Urstad 1997 (1)	P	GP	10	43	0.59	10	39	0.55	NA	NA	NA	NA	NA	NA	NA	NA	NA	NA
Nordstrom 2001 (2)	P	GP	249	50	0.69	218	52	0.67	28.2	166	NA	51	NA	10	2	6	NA	25
Myers 2002 (3)	R	GP	15	36	0.50	15	35	0.53	NA	NA	NA	NA	NA	NA	NA	NA	NA	NA
Olsen 2003 (4)	R	CD	73	67	1.00	26	66	0.95	27.5	NA	NA	NA	NA	100	NA	NA	NA	NA
Kim 2003 (5)	R	CD	240	50	0.75	106	54	0.70	25.0	182	NA	44	NA	NA	NA	NA	NA	NA
Zoungas 2004 (6)	P	CD	355	58	0.79	173	56	0.77	26.6	211	135	48	152	NA	NA	NA	NA	NA
Zureik 2004 (7)	P	GP	583	53	0.72	579	48	0.69	24.9	236	NA	NA	NA	20	4	NA	NA	13
Czarnecka 2005 (8)	P	GP	135	34	0.66	159	37	0.68	25.4	NA	NA	NA	NA	32	4	NA	NA	30
Hegele 2005 (9)	R	GP	59	36	0.78	91	39	0.77	29.2	NA	NA	NA	NA	30	53	NA	NA	15
Riccio 2006 (10)	R	GP	118	68	0.88	51	66	0.84	29.7	180	181	NA	NA	48	57	41	NA	17
Blanco 2006 (11)	R	GP	132	73	0.98	160	72	0.89	NA	NA	NA	NA	NA	77	10	39	NA	16
Polak 2011 (12)	P	CD	584	41	0.64	532	40	0.59	NA	190	114	57	90	30	NA	32	NA	16
Herder 2012_1 (13)	P	GP	1307	56	0.73	1436	57	0.69	25.8	256	NA	55	142	NA	1	NA	6	26
Lindenmaier 2013 (14)	P	CD	24	64	0.78	15	64	0.73	27.3	159	83	52	114	NA	NA	NA	NA	NA
Fromm 2014 (15)	P	CD	122	50	0.88	112	48	0.72	NA	NA	NA	NA	NA	50	11	54	NA	69
Gomez-Sanchez 2015 (16)	R	GP	272	59	0.75	228	62	0.72	27.7	216	135	54	NA	80	28	84	NA	23
Lu 2017 (17)	P	GP	711	73	0.63	888	73	0.60	23.9	NA	124	54	NA	53	20	34	NA	23
van Mil 2019 (18)	P	GP	52	44	0.64	148	41	0.53	41.4	NA	120	47	168	35	20	29	NA	26
Chambless 1997 (19)	P	GP	5552	55	0.66	7289	54	0.60	NA	215	137	53	NA	32	8	NA	NA	26
Chambless 2000 (20)	P	GP	6349	55	0.66	7865	54	0.60	27.3	215	NA	NA	NA	33	9	NA	NA	26

Hoed 2015 (21)	R	GP	4055	62	0.76	5076	61	0.71	26.4	NA	150	49	NA	NA	12	NA	NA	20
Magnusson 2013 (22)	P	GP	1915	58	0.74	2821	58	0.70	25.6	NA	161	52	NA	NA	8	NA	NA	26
Baldassarre 2012 (23)	P	GP	1666	64	0.74	1780	64	0.70	27.3	NA	138	47	NA	NA	26	NA	NA	15
Andersson 2009 (24)	R	GP	474	70	0.88	475	70	0.85	27.1	NA	130	56	NA	NA	12	NA	NA	11
EDIC group 1999 (25)	R	CD	762	36	0.69	705	35	0.66	26.1	187	146	54	91	16	NA	NA	NA	91
Baldassarre 2002 (26)	R	CD	245	51	0.77	314	56	0.70	23.6	246	186	54	125	27	NA	NA	NA	45
Karvonen 2002 (27)	R	CD	505	51	1.00	519	52	0.84	27.7	221	170	51	145	NA	NA	NA	NA	52
Gaenzer 2002 (28)	P	GP	76	41	0.58	26	34	0.44	24.3	196	116	60	136	NA	NA	NA	NA	10
Strohmer 2005 (29)	P	GP	851	49	0.75	348	56	0.76	26.6	NA	NA	NA	NA	51	NA	NA	NA	NA
Astrand 2005 (30)	R	GP	50	46	0.60	58	48	0.58	23.5	NA	NA	NA	NA	NA	NA	NA	NA	NA
de Vries 2005 (31)	R	CD	98	59	0.89	71	54	0.79	27.5	210	NA	58	132	NA	51	NA	NA	NA
Poykko 2006 (32)	R	GP	509	51	0.87	515	52	0.76	27.7	220	137	52	141	NA	52	NA	NA	NA
Cardellini 2007 (33)	R	GP	58	38	0.77	118	35	0.73	29.1	192	116	56	106	NA	NA	NA	NA	33
Fan 2006 (34)	P	GP	231	49	0.71	182	52	0.68	28.6	NA	NA	NA	NA	NA	NA	NA	NA	28
Kawamoto 2007 (35)	R	GP	388	64	0.93	480	70	0.93	NA	NA	112	51	92	NA	27	3	NA	NA
Pertovaara 2007 (36)	P	GP	439	32	0.60	541	32	0.57	24.8	NA	NA	NA	NA	NA	NA	NA	NA	NA
Chang 2007 (37)	P	GP	130	25	0.68	150	25	0.68	21.8	164	NA	58	90	NA	NA	NA	NA	36
Lee 2008 (38)	P	GP	113	25	0.69	143	25	0.68	21.8	165	91	58	91	NA	NA	NA	NA	NA
O’Donnell 2008 (39)	R	GP	496	60	0.64	566	59	0.58	27.7	206	NA	52	135	41	7	NA	10	13
Romero 2008 (40)	R	GP	439	58	0.70	567	58	0.67	NA	NA	NA	NA	NA	NA	2	NA	2	NA
Bertoni 2009 (41)	R	GP	3089	62	0.89	3393	62	0.85	28.2	194	NA	51	132	45	15	NA	NA	37
Jylhävä 2008 (42)	P	GP	704	32	0.59	994	32	0.57	24.9	199	126	50	100	NA	NA	NA	NA	NA
Páramo 2008 (43)	R	GP	311	54	0.73	89	57	0.66	28.0	222	150	49	121	51	16	81	NA	30
Suh 2009 (44)	R	GP	1054	56	0.95	1595	55	0.88	24.6	197	NA	NA	144	34	9	NA	NA	15
Jylhava 2009 (45)	P	GP	891	32	0.59	618	32	0.58	25.1	199	129	48	95	NA	NA	NA	NA	25
Shah 2009 (46)	R	CD	50	19	0.58	79	19	0.52	36.7	188	115	45	148	NA	100	NA	NA	NA

Hyder 2010 (47)	R	GP	929	64	0.89	904	65	0.85	28.0	195	NA	51	NA	45	12	NA	0.3	57
Sojkova 2010 (48)	R	GP	42	72	0.70	31	70	0.60	NA	NA	NA	NA	NA	44	NA	NA	8	NA
Lee 2010 (49)	P	GP	634	66	0.75	1096	63	0.70	23.6	188	NA	51	126	NA	NA	NA	NA	11
Uurtuya 2010 (50)	R	CD	142	56	0.89	170	58	0.73	25.6	183	NA	NA	NA	47	29	NA	NA	22
Liao 2010 (51)	R	GP	482	53	0.64	531	53	0.59	NA	NA	NA	NA	NA	31	10	5	NA	NA
Chumaeva 2010 (52)	R	GP	719	32	0.59	1002	32	0.57	NA	NA	114	50	127	NA	NA	NA	NA	NA
Lin 2010 (53)	R	GP	496	52	0.63	587	52	0.59	24.4	NA	NA	NA	NA	27	8	16	NA	10
Irace 2011 (54)	R	GP	33	52	0.68	15	56	0.72	27.0	210	NA	51	110	NA	NA	NA	NA	NA
Franck 2012 (55)	P	CD	309	61	0.76	173	61	0.69	30.0	NA	104	51	156	NA	100	NA	NA	NA
Rossi 2012 (56)	R	GP	465	45	0.70	546	45	0.70	27.4	NA	139	54	NA	NA	15	NA	NA	16
Janicki-Deverts 2011 (57)	P	GP	1283	25	0.70	1276	25	0.66	27.5	181	NA	52	93	NA	NA	NA	NA	NA
Gomez-Marcos 2012 (58)	P	CD	153	52	0.73	105	55	0.69	27.9	209	131	53	126	NA	NA	80	NA	24
Herder 2 2012 (59)	P	GP	1442	56	0.84	1532	57	0.79	NA	256	170	60	137	NA	1	NA	NA	NA
Cipolli 2012 (60)	R	CD	131	59	0.79	207	57	0.75	30.8	NA	110	51	98	NA	28	NA	NA	9
Su 2012 (61)	R	GP	1203	56	0.74	1487	54	0.66	24.1	203	126	41	118	28	27	NA	5	26
Dahlén 2013 (62)	P	CD	172	61	0.70	83	61	0.67	29.6	183	97	53	NA	NA	NA	NA	NA	16
Itkonen 2013 (63)	R	GP	176	42	0.57	370	42	0.54	26.8	211	113	58	115	NA	NA	NA	NA	NA
Kim 2014 (64)	P	GP	1624	52	0.71	1505	52	0.70	24.4	196	NA	45	135	NA	NA	NA	NA	18
Spartano 2014 (65)	R	GP	12	39	0.49	13	37	0.46	23.6	186	117	69	NA	NA	NA	NA	NA	NA
Kedenko 2014 (66)	P	GP	1107	49	0.76	663	56	0.77	26.8	229	NA	60	NA	56	3	NA	NA	19
Lee 2014 (67)	P	GP	3019	62	0.82	4535	61	0.77	24.3	188	NA	48	128	NA	NA	NA	NA	15
Rosvall 2015 (68)	P	GP	1522	56	0.77	2212	56	0.74	25.5	NA	160	54	NA	NA	6	NA	NA	19
Jones 2015 (69)	R	GP	2991	62	0.90	3356	62	0.85	28.3	194	NA	51	NA	NA	13	NA	NA	13
Persson 2015 (70)	P	GP	1653	65	0.74	1777	65	0.70	26.8	NA	136	47	117	NA	26	NA	NA	15
Ceponiene 2015 (71)	P	GP	168	48	0.66	212	48	0.61	26.1	NA	151	63	NA	48	29	NA	NA	NA

Gómez-Marcos 2015 (72)	P	CD	71	61	0.78	41	62	0.72	30.0	200	122	47	153	NA	NA	NA	2	22
Shen 2015 (73)	R	GP	95	47	0.54	88	44	0.52	23.2	165	97	51	86	NA	NA	NA	NA	NA
Hakulinen 2016 (74)	P	GP	1018	32	0.59	1247	32	0.57	25.1	NA	126	NA	NA	NA	NA	NA	NA	NA
Shin 2017 (75)	R	GP	723	46	0.57	956	45	0.52	23.7	190	110	52	116	15	11	NA	NA	NA
Thorsson 2017 (76)	R	GP	4843	56	0.78	5700	57	0.73	27.4	NA	NA	NA	NA	NA	6	NA	6	23
Sigurdsson 1993 (77)	R	GP	2629	54	0.77	2719	54	0.72	27.8	NA	NA	NA	NA	NA	5	NA	6	21
Herder 2012_2 (59)	P	GP	2214	59	0.79	2981	60	0.73	26.9	NA	NA	NA	NA	NA	8	NA	6	25
Yasuda 2020 (78)	R	CD	56	69	1.10	16	66	1.00	NA	NA	110	49	122	69	NA	NA	NA	33
Holwerda 2019 (79)	R	GP	52	35	0.44	41	40	0.44	28.3	173	101	53	88	25	NA	NA	NA	19
Gómez-Marcos 2018 (80)	R	GP	1456	62	0.73	898	62	0.73	28.6	225	141	48	123	73	34	67	NA	28
Hanna 2018 (81)	P	GP	1304	50	0.73	1722	40	0.71	26.8	180	NA	46	NA	21	10	14	NA	39
Basu 2018 (82)	P	CD	269	42	0.68	148	39	0.61	26.9	189	115	55	74	35	100	NA	NA	15
Sturlaugsdottir 2017 (83)	R	GP	3204	50	0.76	3320	50	0.71	27.5	203	126	56	88	34	4	NA	3	21
Ma 2017 (84)	R	CD	512	59	0.75	958	57	0.70	22.8	196	113	59	118	NA	NA	NA	NA	22
Rosfors 1998 (85)	P	GP	90	68	0.89	92	71	0.80	NA	NA	NA	NA	NA	27	8	NA	30	NA
Kapiotis 2006 (86)	R	GP	75	15	0.40	70	15	0.37	35.4	184	105	44	117	NA	NA	NA	NA	NA
Nyasordzi 2020 (87)	P	GP	120	23	0.57	145	24	0.55	23.5	NA	NA	NA	NA	NA	NA	NA	NA	NA
Maloberti 2021 (88)	R	GP	273	42	0.60	106	43	0.60	NA	190	115	56	97	NA	NA	NA	NA	36
Karikkineth 2020 (89)	P	GP	512	65	0.76	555	61	0.70	26.7	NA	110	54	108	49	13	54	NA	NA
Wang 2021 (90)	R	GP	32	48	0.55	40	50	0.60	24.4	NA	NA	NA	NA	NA	NA	NA	NA	NA

P: prospective; R: retrospective; GT: general population, CD: chronic cardiovascular disease population; CCA IMT: common carotid artery intima-media thickness; BMI: body mass index; TC: total cholesterol; HT: hypertension; DM: diabetes mellitus; DLD: dyslipidemia; CAD: coronary artery disease.

Supplementary Table S2. Clinical and demographic characteristics of subjects included in studies where intima-media thicknesses of carotid artery bifurcation were measured.

Author + Year	Men, n	Age	Bif IMT mean	Women, n	Age	Bif IMT mean	BMI kg/m2	TC, mg/dL	LDL mg/dL	HDL mg/dL	TG mg/dL	HT, %	DM, %	Smokers, %
Chambless 1997	5552	54	0.892	7289	54	0.786	NA	214	137	53	NA	NA	NA	NA
Chambless 2000	6349	54	0.902	7865	55	0.782	27	215	NA	NA	NA	33	9	26
den Hoed 2015	4055	61	1.251	5076	62	1.134	26.4	NA	150	51	NA	NA	12	20
Magnusson 2013	1915	58	1.390	2821	58	1.240	25.6	NA	161	52	NA	NA	8	26
Baldassarre 2012	1666	64	1.163	1780	64	1.017	27.2	NA	138	47	NA	NA	26	15
Andersson 2009	474	70	0.997	475	70	0.950	27.1	NA	130	56	NA	NA	12	11
Baldassarre 2002	245	56	1.010	314	51	0.900	23.6	246	186	52	308	27	NA	45
Karvonen 2002	505	52	0.968	519	51	0.915	27.7	220	137	52	141	NA	NA	52
Poykko 2006	509	52	0.965	515	51	0.915	27.6	220	137	52	141	NA	NA	NA
Shah 2009	50	19	0.550	79	19	0.510	36.7	188	115	45	148	NA	NA	NA
Liao 2010	482	53	0.670	531	53	0.630	NA	NA	NA	NA	NA	31	10	NA
Lin 2010	496	52	0.670	587	52	0.620	24.6	NA	NA	NA	NA	27	8	10
Persson 2015	1653	65	1.157	1777	65	1.020	26.8	NA	136	47	123	NA	26	15
Shin 2017	723	45	0.814	956	46	0.703	23.7	190	110	52	116	15	11	NA

Bif IMT: bifurcation carotid artery intima media thickness; BMI: body mass index; TC: total cholesterol; HT: hypertension; DM: diabetes mellitus.

Supplementary Table S3. Clinical and demographic characteristics of subjects included in studies where intima-media thicknesses of the internal carotid artery were measured.

Author + Year	Men, n	Age	ICA IMT mean	Women, n	Age	ICA IMT mean	BMI kg/m2	TC, mg/dL	LDL mg/dL	HDL mg/dL	TG mg/dL	HT, %	DM, %	Smokers, %
Chambless 1997	5552	55	0.740	7289	54	0.658	NA	215	137	53	NA	NA	NA	NA
Chambless 2000	6349	55	0.741	7865	54	0.662	27.4	215	NA	NA	NA	33	9	14
EDIC research group 1999	762	36	0.708	705	35	0.640	26.1	187	115	54	91	16	0	7
Baldassarre 2002	245	51	0.780	314	56	0.680	23.6	246	186	52	308	27	0	14
Karvonen 2002	505	51	0.824	519	52	0.729	27.7	220	137	52	141	0	0	18
Strohmer 2005	851	49	0.822	348	56	0.815	26.6	NA	NA	NA	NA	51	NA	NA
Poykko 2006	509	51	0.820	515	52	0.738	27.6	220	137	52	141	NA	NA	NA
O'Donnell 2008	496	60	0.630	566	59	0.490	27.7	206	NA	52	135	41	7	14
Romero 2008	439	58	2.110	567	58	1.390	NA	NA	NA	NA	NA	NA	2	1
Bertoni 2009	3089	62	1.140	3393	62	1.010	28.2	194	NA	51	132	45	15	16
Shah 2009	50	19	0.480	79	19	0.420	36.7	188	115	45	148	NA	NA	NA
Hyder 2010	929	64	1.150	904	65	1.020	28.0	195	NA	51	NA	45	12	24
Liao 2010	482	53	0.540	531	53	0.480	NA	NA	NA	NA	NA	31	10	NA
Lin 2010	496	52	0.530	587	52	0.480	24.2	NA	NA	NA	NA	27	8	1
Kedenko 2014	1107	49	0.827	663	56	0.826	26.8	229	NA	60	NA	56	3	5
Shin 2017	723	46	0.508	956	45	0.444	23.7	1905	110	52	116	15	11	NA
Basu 2018	269	42	0.836	148	39	0.693	26.9	189	115	55	78	35	100	5

ICA IMT: internal carotid artery intima media thickness; BMI: body mass index; TC: total cholesterol; HT: hypertension; DM: diabetes mellitus.

Supplementary Table S4. Meta-regressions analysis for studies included measurements of intima-media thickness in common carotid artery.

**Variable**	**Z - Value**	**P - Value**
**Age**	0.35	0.728
**BMI**	1.92	0.055
**Total cholesterol**	1.64	0.101
**LDL**	1.48	0.140
**HDL**	-1.00	0.316
**Triglycerides**	0.91	0.365
**Hypertension**	-1.48	0.140
**Diabetes**	1.89	0.058
**Dyslipidemia**	0.43	0.667
**CAD**	1.31	0.189
**Smokers**	0.16	0.875

BMI: body mass index; CAD coronary artery disease; HDL: high density lipoproteins; LDL: low density lipoproteins.

Supplementary Table S5. Meta-regressions analysis for general population studies.

**Variable**	**Z - Value**	**P - Value**
**Age**	-0.05	0.962
**BMI**	1.44	0.150
**Total cholesterol**	-0.25	0.801
**LDL**	-0.41	0.679
**HDL**	-1.57	0.116
**Triglycerides**	2.53	**0.012**
**Hypertension**	-0.61	0.544
**Dyslipidemia**	0.24	0.814
**Diabetes**	0.02	0.987
**CAD**	1.42	0.155
**Smokers**	-1.21	0.225

BMI: body mass index; CAD coronary artery disease; HDL: high density lipoproteins; LDL: low density lipoproteins.

Supplementary Table S6. Meta-regressions analysis for cardiovascular disease studies.

Variable	Z - Value	P - Value
**Age**	0.08	0.337
**BMI**	0.09	0.932
**Total cholesterol**	-0.96	0.337
**LDL**	1.71	0.090
**HDL**	0.49	0.625
**Triglycerides**	-1.34	0.180
**Hypertension**	-1.09	0.277
**Diabetes**	-1.21	0.225
**Smokers**	-0.55	0.581

BMI: body mass index; HDL: high density lipoproteins; LDL: low density lipoproteins.

Supplementary Table S7. Meta-regressions analysis for studies included intima-media thickness measurement of carotid bifurcation.

Variable	Z - Value	P - Value
**Age**	-0.02	0.985
**BMI**	-0.12	0.901
**Total cholesterol**	0.82	0.414
**LDL**	0.12	0.902
**HDL**	0.85	0.396
**Triglycerides**	0.76	0.445
**Hypertension**	0.67	0.503
**Diabetes**	-0.36	0.719
**Smokers**	1.05	0.294

BMI: body mass index; HDL: high density lipoproteins; LDL: low density lipoproteins.

Supplementary Table S8. Meta-regressions analysis for studies included intima-media thickness measurement of internal carotid artery.

Variable	Z - Value	P - Value
**Age**	0.39	0.698
**BMI**	0.28	0.776
**Total cholesterol**	-0.45	0.653
**LDL**	1.88	0.060
**HDL**	0.00	0.999
**Triglycerides**	0.84	0.400
**Hypertension**	-1.06	0.288
**Diabetes**	0.01	0.989
**Smokers**	0.21	0.836

BMI: body mass index; HDL: high density lipoproteins; LDL: low density lipoproteins.

Supplemental Table S9, Quality assessment with Newcastle-Ottawa scale of the included studies.

Author, year	SELECTION				COMPARABILITY	OUTCOME			
	Representativeness of the Exposed Cohort	Selection of the Non-Exposed Cohort	Ascertainment of Exposure	Demonstration That Outcome of Interest Was Not Present at Start of Study		Assessment of Outcome	Enough Follow-Up Long for Outcomes to Occur	Adequacy of Follow Up of Cohorts	Quality
Sigurdsson 1993	**	*	-	-	*	*	-	-	**5**
Jensen-Urstad 1997	**	*	*	-	-	**	*	-	**7**
Chambless 1997	**	*	*	-	*	*	*	-	**7**
Rosfors 1998	**	*	*	-	*	**	-	-	**7**
EDIC group 1999	**	*	*	-	*	*	*	-	**7**
Chambless 2000	**	*	*	-	*	*	*	-	**7**
Nordstrom 2001	**	*	*	-	*	*	*	-	**7**
Elhadd 2001	**	*	*	-	*	*	-	-	**6**
Baldassarre 2002	**	*	*	-	*	*	*	-	**7**
Myers 2002	**	*	*	-	-	*	*	-	**6**
Gaenzer 2002	*	*	*	-	-	*	*	-	**5**
Karvonen 2002	**	*	*	-	*	*	*	-	**6**
Olsen 2003	**	*	*	-	-	**	*	-	**7**

Kim 2003	-	*	*	-	*	**	*	-	**6**
Zureik 2004	-	*	*	-	*	**	*	-	**6**
Zoungas 2004	-	*	*	-	*	**	*	-	**6**
Strohmer 2005	-	*	*	-	*	*	*	-	**5**
Czarnecka 2005	**	*	*	-	*	*	*	-	**7**
Åstrand 2005	**	*	*	-	-	*	*	-	**6**
De Vries 2005	**	*	*	-	*	*	*	-	**7**
Hegele 2005	**	*	*	-	*	*	*	-	**6**
Fan 2006	*	*	*	-	*	*	*	-	**6**
Blanco 2006	**	*	*	-	-	**	*	-	**7**
Pöykkö 2006	**	*	*	-	*	**	-	-	**7**
Riccio 2006	*	*	*	-	-	*	*	-	**5**
Kapiotis 2006	**	*	*	-	-	**	-	-	**6**
Chang 2007	**	*	*	-	-	*	*	-	**6**
Kawamoto 2007	*	*	*	-	*	*	*	-	**6**
Pertovaara 2007	**	*	*	-	-	*	*	-	**6**
Cardellini 2007	*	*	*	-	*	*	-	-	**5**
O'Donnell 2008	**	*	*	-	*	*	*	-	**6**

Jylhävä 2008	**	*	*	-	*	*	-	-	**6**
Páramo 2008	-	*	*	-	*	*	*	-	**5**
Romero 2008	*	*	*	-	*	**	*	-	**7**
Lee 2008	*	*	*	-	*	-	-	-	**4**
Bertoni 2009	**	*	*	-	*	*	*	-	**7**
Shah 2009	*	*	*	-	*	**	*	-	**7**
Andersson 2009	**	*	*	-	*	*	*	-	**7**
Jylhävä 2009	**	*	*	-	*	*	*	-	**7**
Suh 2009	*	*	*	-	*	*	*		**6**
Lin 2010	**	*	*	-	*	**	-	-	**7**
Sojkova 2010	**	*	*	-	*	*	*	-	**7**
Hydera 2010	**	*	*	-	*	*	*	-	**6**
Chumaeva 2010	-	*	*	-	*	**	*	-	**6**
Uurtuya 2010	**	*	*	-	*	*	*	-	**7**
Liao 2010	**	*	*	-	*	*	*	-	**6**
Lee 2010	*	*	*	-	*	*	-	-	**5**
Irace 2011	-	*	*	-	*	**	*	-	**6**
Janicki-Deverts 2011	**	*	*	-	*	*	*	-	**7**

Polak 2011	**	*	*	-	*	*	*	-	**7**
Baldassarre 2012	**	*	*	-	*	*	*	-	**6**
Herder 2012	**	*	*	-	*	**	-	-	**7**
Rossi 2012	**	*	*	-	*	*	*	-	**6**
Cipolli 2012	-	*	*	-	*	*	*	-	**5**
Gomez-Marcos 2012	*	*	*	-	*	**	*	-	**7**
Herder 2012	**	*	*	-	*	*	*	-	**7**
Herder 2012	**	*	*	-	*	*	*	-	**6**
Franck 2012	-	*	*	-	*	*	*	-	**5**
Su 2012	**	*	*	-	*	*	-	-	**6**
Dahlén 2013	*	*	*	-	*	*	-	-	**5**
Magnusson 2013	**	*	*	-	*	*	*	-	**7**
Itkonen 2013	-	*	*	-	*	*	*	-	**5**
Lindenmaier 2013	**	*	*	-	-	**	*	-	**7**
Fromm 2014	**	*	*	-	-	*	*	-	**6**
Kim 2014	**	*	*	-	*	*	-	-	**6**
Kedenko 2014	-	*	*	-	*	**	*	-	**6**

Spartano 2014	**	*	*	-	*	*	*	-	**7**
Lee 2014	*	*	*	-	*	*	*	-	**6**
Shen 2015	**	*	*	-	*	*	*	-	**6**
Ceponiene 2015	**	*	*	-	*	*	-	-	**6**
Persson 2015	**	*	*	-	*	**	-	-	**7**
Gomez-Sanchez 2015	**	*	*	-	*	*	*	-	**6**
Rosvall 2015	*	*	*	-	*	*	*	-	**6**
Gómez-Marcos 2015	*	*	*	-	-	**	*	-	**6**
Den Hoed 2015	**	*	*	-	*	*	-	-	**6**
Jones 2015	**	*	*	-	*	*	*	-	**7**
Hakulinen 2016	**	*	*	-	*	*	*	-	**7**
Thorsson 2017	*	*	*	-	*	**	-	-	**6**
Ma 2017	-	*	*	-	*	**	*	-	**6**
Shin 2017	**	*	*	-	*	*	*	-	**7**
Sturlaugsdottir 2017	**	*	*	-	-	*	*	-	**6**
Lu 2017	**	*	*	-	*	*	*	-	**7**
Basu 2018	*	*	*	-	*	*	-	-	**5**

Hanna 2018	*	*	*	-	*	*	-	-	**5**
Gomez-Marcos 2018	-	*	*	-	*	**	*	-	**6**
Holwerda 2019	**	*	*	-	*	*	*	-	**7**
Van Mil 2019	-	*	*	-	*	*	*	-	**5**
Karikkineth 2020	**	*	*	-	*	*	-	-	**6**
Nyasordzi 2020	**	*	*	-	*	*	-	-	**6**
Yasuda 2020	*	*	*	-	-	*	*	-	**5**
Maloberti 2021	*	-	*	-	*	**	-	-	**5**

Study name	Subgroup within study	Std diff in means	Standard error	Variance	Lower limit	Upper limit	Z-Value	p-Value
		**Statistics for each study**						
ZHENZHEN WANG 2021	< 50	-0,837	0,247	0,061	-1,322	-0,353	-3,387	0,001
CHRISTOPHER W. MYERS 2002	< 50	-0,300	0,367	0,135	-1,020	0,420	-0,817	0,414
D Czarnecka 2005	< 50	-0,059	0,117	0,014	-0,289	0,170	-0,507	0,612
Alessandro Maloberti 2021	< 50	0,000	0,114	0,013	-0,224	0,224	0,000	1,000
Hoo Sun Chang 2007	< 50	0,000	0,120	0,014	-0,235	0,235	0,000	1,000
Isabelle Anne Rossi 2012	< 50	0,000	0,063	0,004	-0,124	0,124	0,000	1,000
Seth W. Holwerda 2019	< 50	0,000	0,209	0,044	-0,409	0,409	0,000	1,000
J. Jylha¨va¨ 2009	< 50	0,017	0,052	0,003	-0,085	0,120	0,329	0,742
Christian Hakulinen 2016	< 50	0,036	0,042	0,002	-0,047	0,119	0,852	0,394
Suvi T Itkonen 2013	< 50	0,044	0,092	0,008	-0,136	0,223	0,477	0,634
Robert A. Hegele 2005	< 50	0,059	0,167	0,028	-0,268	0,387	0,356	0,722
Håkan Åstrand 2005	< 50	0,134	0,193	0,037	-0,245	0,512	0,692	0,489
Yoo Jung Lee 2008	< 50	0,139	0,126	0,016	-0,108	0,386	1,101	0,271
Hao Shen 2015	< 50	0,148	0,148	0,022	-0,142	0,439	1,001	0,317
David B. Hanna 2018	< 50	0,199	0,037	0,001	0,127	0,271	5,412	0,000
Nadja Chumaeva 2010	< 50	0,225	0,049	0,002	0,129	0,321	4,589	0,000
J. Jylhävä 2008	< 50	0,225	0,049	0,002	0,128	0,322	4,556	0,000
Nicole L. Spartano 2014	< 50	0,316	0,403	0,162	-0,473	1,106	0,785	0,433
M. Pertovaara 2007	< 50	0,317	0,065	0,004	0,190	0,444	4,905	0,000
Marina Cardellini 2007	< 50	0,331	0,161	0,026	0,014	0,647	2,049	0,040
DENISE JANICKI-DEVERTS 2011	< 50	0,347	0,040	0,002	0,269	0,426	8,723	0,000
Ran Sturlaugsdottir 2017	< 50	0,357	0,025	0,001	0,308	0,406	14,290	0,000
Juliana Nyasordzi 2020	< 50	0,365	0,124	0,015	0,121	0,609	2,936	0,003
Jinyoung Shin 2017	< 50	0,428	0,050	0,002	0,330	0,526	8,590	0,000
NIH-PA Author Manuscript 1999	< 50	0,483	0,053	0,003	0,379	0,587	9,107	0,000
K. Jensen-Urstad 1997	< 50	0,500	0,454	0,206	-0,390	1,390	1,101	0,271
Joseph F. Polak, 2011	< 50	0,572	0,061	0,004	0,452	0,692	9,350	0,000
Amy S. Shah, 2009	< 50	0,611	0,185	0,034	0,249	0,973	3,308	0,001
Arpita Basu 2018	< 50	0,627	0,105	0,011	0,422	0,832	5,993	0,000
Indre Ceponiene 2015	< 50	0,682	0,106	0,011	0,473	0,890	6,417	0,000
Annette Fromm 2014	< 50	0,800	0,136	0,018	0,533	1,067	5,883	0,000
Stylianos Kapiotis 2006	< 50	0,939	0,175	0,031	0,596	1,282	5,362	0,000
Stefanie R. van Mil 2019	< 50	1,053	0,170	0,029	0,720	1,385	6,208	0,000
Hannes Gaenzer 2002	< 50	1,117	0,240	0,058	0,646	1,587	4,647	0,000
**< 50 Overall**		0,294	0,067	0,004	0,163	0,424	4,416	0,000
Concetta Irace 2011	> 50	-0,319	0,313	0,098	-0,933	0,295	-1,019	0,308
B. STROHMER 2005	> 50	-0,082	0,064	0,004	-0,207	0,042	-1,295	0,195
Lyudmyla Kedenko 2014	> 50	-0,079	0,049	0,002	-0,175	0,018	-1,599	0,110
Manuel A. Gomez-Marcos 2018	> 50	-0,000	0,042	0,002	-0,083	0,083	-0,000	1,000
Kawamoto R. 2007	> 50	0,000	0,068	0,005	-0,134	0,134	0,000	1,000
Jose R. Romero 2008	> 50	0,003	0,064	0,004	-0,122	0,127	0,044	0,965
Marcel den Hoed 2015	> 50	0,015	0,021	0,000	-0,026	0,057	0,731	0,465
M. Magnusson 2013	> 50	0,016	0,030	0,001	-0,042	0,074	0,552	0,581
J. Andersson 2009	> 50	0,022	0,065	0,004	-0,105	0,150	0,347	0,729
Baldassarre 2012	> 50	0,024	0,034	0,001	-0,043	0,090	0,693	0,489
Lloyd E. Chambless 2000	> 50	0,045	0,017	0,000	0,012	0,078	2,652	0,008
Marit Herder 2012	> 50	0,056	0,038	0,001	-0,019	0,131	1,475	0,140
SOPHIA ZOUNGAS 2004	> 50	0,128	0,093	0,009	-0,053	0,310	1,384	0,166
Elsa M Dahlén 2013	> 50	0,178	0,134	0,018	-0,084	0,441	1,333	0,182
Cheryl K. Nordstrom 2001	> 50	0,180	0,093	0,009	-0,002	0,362	1,937	0,053
Yuyan Lu 2017	> 50	0,204	0,050	0,003	0,105	0,303	4,041	0,000
M. Rosvall 2015	> 50	0,210	0,033	0,001	0,144	0,275	6,283	0,000
Joseph A. Hydera 2010	> 50	0,210	0,047	0,002	0,118	0,302	4,484	0,000
Alain G. Bertoni 2009	> 50	0,211	0,025	0,001	0,162	0,260	8,451	0,000
Silvia A Riccio 2006	> 50	0,240	0,168	0,028	-0,089	0,570	1,429	0,153
Miranda R Jones 2015	> 50	0,262	0,025	0,001	0,213	0,312	10,382	0,000
Mina Suh 2009	> 50	0,285	0,040	0,002	0,207	0,363	7,145	0,000
Hsiu-Fen Lin 2010	> 50	0,286	0,061	0,004	0,166	0,406	4,670	0,000
Marit Herder 2 2012	> 50	0,311	0,037	0,001	0,238	0,383	8,415	0,000
S. M. PO¨YKKO 2006	> 50	0,320	0,063	0,004	0,197	0,444	5,091	0,000
Amy Z Fan 2006	> 50	0,324	0,100	0,010	0,129	0,520	3,249	0,001
Leticia Gomez-Sanchez 2015	> 50	0,328	0,090	0,008	0,151	0,505	3,630	0,000
Masayuki Yasuda 2020	> 50	0,333	0,285	0,081	-0,225	0,892	1,170	0,242
Soo Kyung Kim 2003	> 50	0,334	0,117	0,014	0,104	0,564	2,844	0,004
Manuel A Gomez-Marcos 2012	> 50	0,337	0,128	0,016	0,087	0,587	2,640	0,008
MH Olsen 2003	> 50	0,337	0,230	0,053	-0,113	0,787	1,468	0,142
Jonas Persson 2015	> 50	0,359	0,034	0,001	0,291	0,426	10,418	0,000
Ajoy C. Karikkineth 2020	> 50	0,363	0,062	0,004	0,242	0,485	5,883	0,000
Young-Hoon Lee 2010	> 50	0,373	0,050	0,003	0,274	0,471	7,411	0,000
NICLAS FRANCK 2012	> 50	0,374	0,096	0,009	0,186	0,561	3,905	0,000
J . KARVONEN 2002	> 50	0,374	0,063	0,004	0,251	0,498	5,935	0,000
Tamas J Lindenmaier 2013	> 50	0,377	0,350	0,122	-0,309	1,063	1,078	0,281
José A. Páramo 2008	> 50	0,389	0,121	0,015	0,151	0,626	3,211	0,001
Hui Ma 2017	> 50	0,392	0,055	0,003	0,284	0,500	7,103	0,000
Mahmoud Zureik 2004	> 50	0,399	0,059	0,004	0,283	0,515	6,734	0,000
Young-Hoon Lee 2014	> 50	0,404	0,024	0,001	0,357	0,450	17,022	0,000
Shuumarjav Uurtuya 2010	> 50	0,409	0,115	0,013	0,184	0,634	3,558	0,000
Bolli Thorsson 2017	> 50	0,411	0,020	0,000	0,372	0,450	20,820	0,000
Yi-Chu Liao 2010	> 50	0,434	0,064	0,004	0,309	0,559	6,816	0,000
Herder M 2012	> 50	0,449	0,028	0,001	0,393	0,505	15,812	0,000
Francisco Blanco 2006	> 50	0,450	0,119	0,014	0,217	0,683	3,780	0,000
Ta-Chen Su, 2012	> 50	0,450	0,039	0,002	0,373	0,527	11,470	0,000
Stefan Rosfors 1998	> 50	0,451	0,150	0,023	0,156	0,745	3,002	0,003
Sigurdsson E 1993	> 50	0,453	0,028	0,001	0,399	0,508	16,366	0,000
Christopher J. O'Donnell 2008	> 50	0,462	0,062	0,004	0,339	0,584	7,406	0,000
Jitka Sojkova 2010	> 50	0,500	0,240	0,058	0,029	0,971	2,080	0,038
Manuel Ángel Gómez?Marcos 2015	> 50	0,519	0,199	0,040	0,129	0,910	2,608	0,009
Rindert de Vries 2005	> 50	0,601	0,159	0,025	0,289	0,914	3,777	0,000
Lloyd E. Chambless 1997	> 50	0,728	0,018	0,000	0,692	0,764	39,598	0,000
Jinyoung Kim 2014	> 50	2,500	0,048	0,002	2,406	2,594	52,369	0,000
Baldassarre D. 2002	> 50	3,298	0,130	0,017	3,043	3,554	25,299	0,000
José Alexandre Addeo Cipolli 2012	> 50	4,000	0,190	0,036	3,627	4,373	21,043	0,000
		0,422	0,049	0,002	0,325	0,519	8,527	0,000

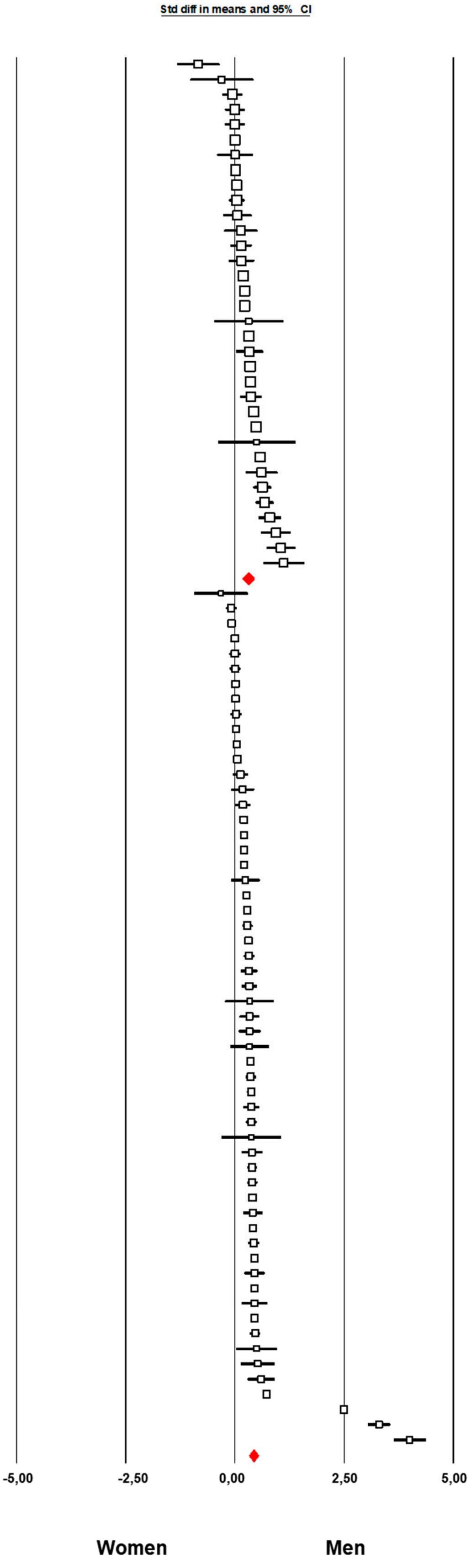

Supplementary Figure S1. Forest plots of common carotid artery intima-media thickness (CCA IMT) difference in men and women in subjects younger and older than 50 years old. Difference in IMT was evaluated with the standardized difference in means (SMD) between men and women. The diamond represents the estimated overall effect, while the squares represent each study with 95% CI.

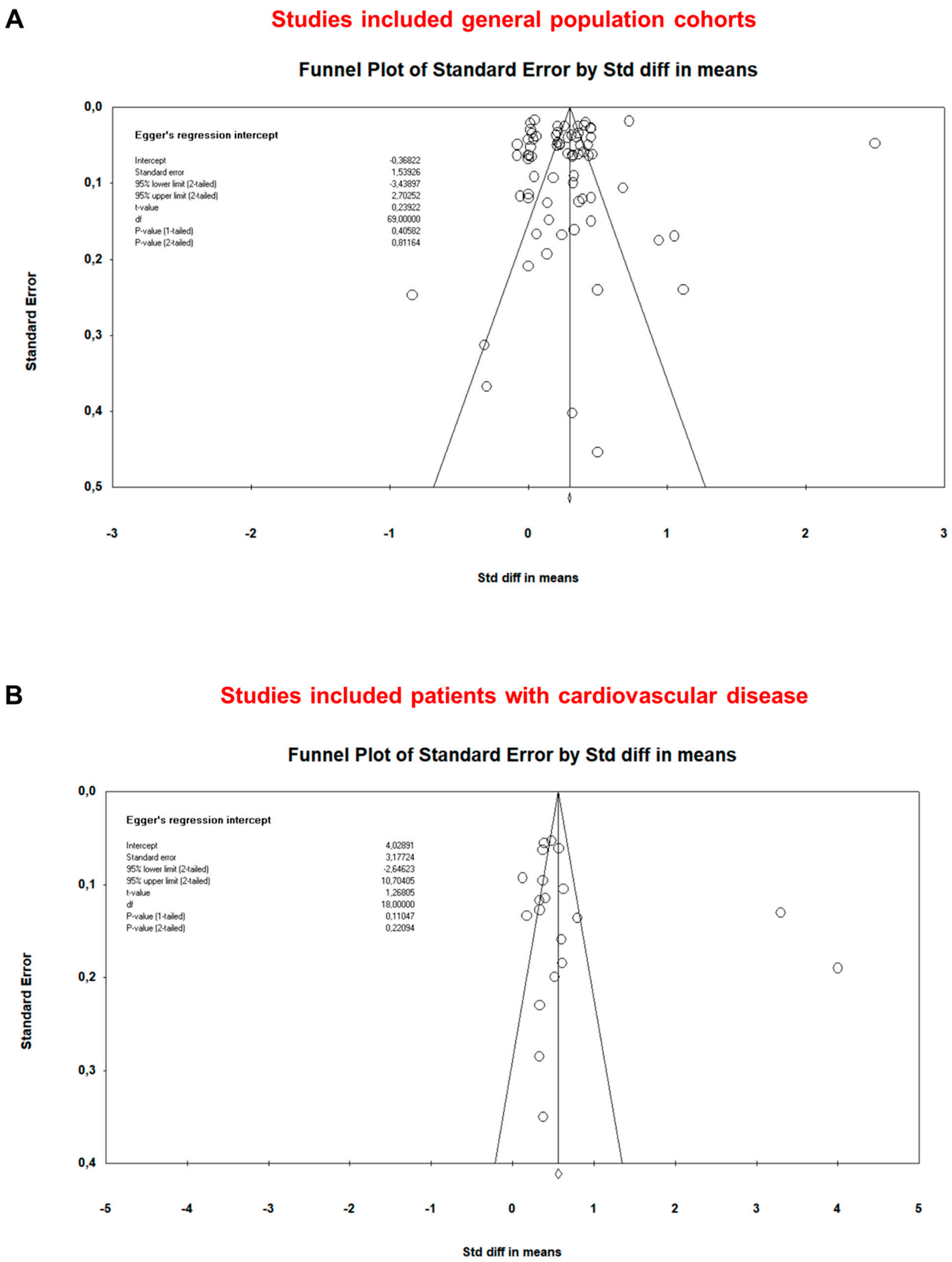

Supplementary Figure S2. Funnel plots of effect size versus standard error for studies measured intima-media thickness of common carotid artery. (**A**) Studies included general population cohorts. (**B**) Studies included cardiovascular disease patients.

A Studies including carotid artery bifurcation measurements

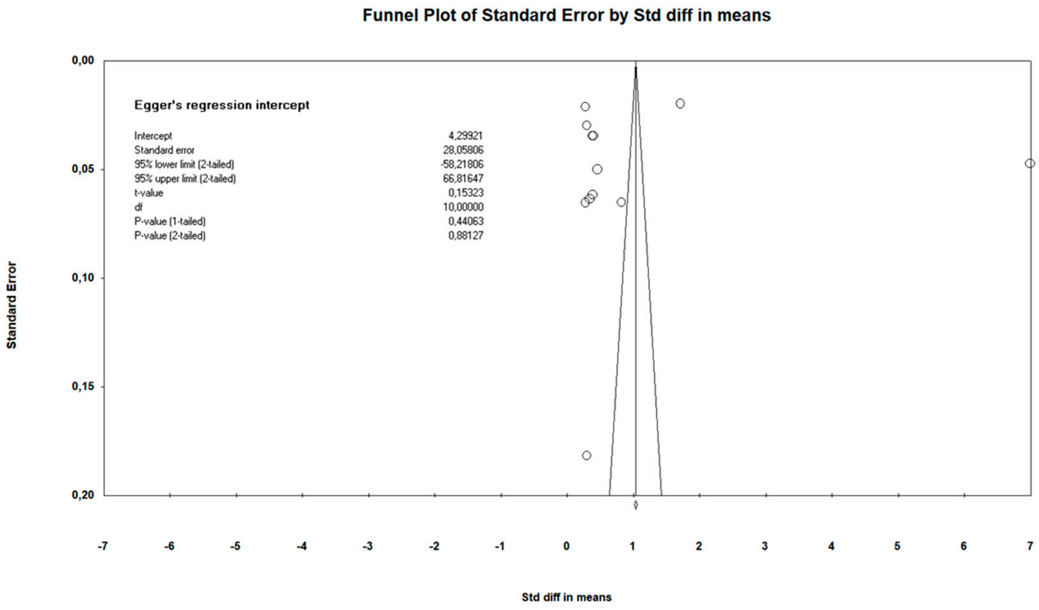

B Studies including internal carotid artery measurements

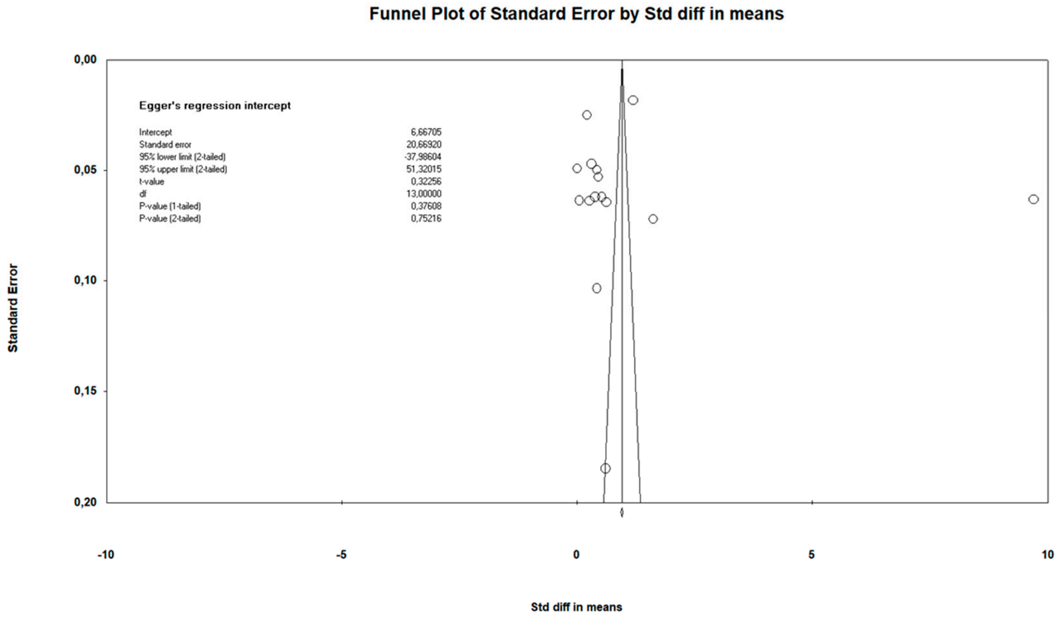

Supplementary Figure S3. Funnel plots of effect size versus standard error for studies measured intima-media thickness of carotid artery bifurcation (**A**) and internal carotid artery (**B**).

References

1. Jensen-Urstad K, Rosfors S. A methodological study of arterial wall function using ultrasound technique. Clin Physiol. 1997;17(6):557-67.
2. Nordstrom CK, Dwyer KM, Merz CN, Shircore A, Dwyer JH. Work-related stress and early atherosclerosis. Epidemiology. 2001;12(2):180-5.
3. Myers CW, Farquhar WB, Forman DE, Williams TD, Dierks DL, Taylor JA. Carotid distensibility characterized via the isometric exercise pressor response. Am J Physiol Heart Circ Physiol. 2002;283(6):H2592-8.
4. Olsen MH, Hjerkinn E, Wachtell K, Hoieggen A, Bella JN, Nesbitt SD, et al. Are left ventricular mass, geometry and function related to vascular changes and/or insulin resistance in long-standing hypertension? ICARUS: a LIFE substudy. J Hum Hypertens. 2003;17(5):305-11.
5. Kim SK, Kim HJ, Hur KY, Choi SH, Ahn CW, Lim SK, et al. Visceral fat thickness measured by ultrasonography can estimate not only visceral obesity but also risks of cardiovascular and metabolic diseases. Am J Clin Nutr. 2004;79(4):593-9.
6. Zoungas S, Branley P, Kerr PG, Ristevski S, Muske C, Demos L, et al. Atherosclerosis and folic acid supplementation trial in chronic renal failure: baseline results. Nephrology (Carlton). 2004;9(3):130-41.
7. Zureik M, Galan P, Bertrais S, Mennen L, Czernichow S, Blacher J, et al. Effects of long-term daily low-dose supplementation with antioxidant vitamins and minerals on structure and function of large arteries. Arterioscler Thromb Vasc Biol. 2004;24(8):1485-91.
8. Czarnecka D, Kawecka-Jaszcz K, Stolarz K, Olszanecka A, Dembinska-Kiec A, Kiec-Wilk B. Ambulatory blood pressure, left ventricular mass and vascular phenotypes in relation to the endothelial nitric oxide synthase gene Glu298Asp and intron 4 polymorphisms in a population-based family study. J Hum Hypertens. 2005;19(5):413-20.
9. Hegele RA, Al-Shali KZ, House AA, Hanley AJ, Harris SB, Mamakeesick M, et al. Disparate associations of a functional promoter polymorphism in PCK1 with carotid wall ultrasound traits. Stroke. 2005;36(12):2566-70.
10. Riccio SA, House AA, Spence JD, Fenster A, Parraga G. Carotid ultrasound phenotypes in vulnerable populations. Cardiovasc Ultrasound. 2006;4:44.
11. Blanco F, Gil P, Arco CD, Saez T, Aguilar R, Lara I, et al. Association of clinic and ambulatory blood pressure with vascular damage in the elderly: the EPICARDIAN study. Blood Press Monit. 2006;11(6):329-35.
12. Polak JF, Backlund JY, Cleary PA, Harrington AP, O'Leary DH, Lachin JM, et al. Progression of carotid artery intima-media thickness during 12 years in the Diabetes Control and Complications Trial/Epidemiology of Diabetes Interventions and Complications (DCCT/EDIC) study. Diabetes. 2011;60(2):607-13.
13. Herder M, Arntzen KA, Johnsen SH, Mathiesen EB. The metabolic syndrome and progression of carotid atherosclerosis over 13 years. The Tromso study. Cardiovasc Diabetol. 2012;11:77.
14. Lindenmaier TJ, Buchanan DN, Pike D, Hartley T, Reid RD, Spence JD, et al. One, two and three-dimensional ultrasound measurements of carotid atherosclerosis before and after cardiac rehabilitation: preliminary results of a randomized controlled trial. Cardiovasc Ultrasound. 2013;11:39.
15. Fromm A, Haaland OA, Naess H, Thomassen L, Waje-Andreassen U. Risk factors and their impact on carotid intima-media thickness in young and middle-aged ischemic stroke patients and controls: the Norwegian Stroke in the Young Study. BMC Res Notes. 2014;7:176.
16. Gomez-Sanchez L, Garcia-Ortiz L, Recio-Rodriguez JI, Patino-Alonso MC, Agudo-Conde C, Rigo F, et al. Leukocyte subtype counts and its association with vascular structure and function in adults with intermediate cardiovascular risk. MARK study. PLoS One. 2015;10(4):e0119963.
17. Lu Y, Zhu M, Bai B, Chi C, Yu S, Teliewubai J, et al. Comparison of Carotid-Femoral and Brachial-Ankle Pulse-Wave Velocity in Association With Target Organ Damage in the Community-Dwelling Elderly Chinese: The Northern Shanghai Study. J Am Heart Assoc. 2017;6(2).

18. van Mil SR, Biter LU, van de Geijn GJM, Birnie E, Dunkelgrun M, Ijzermans JNM, et al. The effect of sex and menopause on carotid intima-media thickness and pulse wave velocity in morbid obesity. Eur J Clin Invest. 2019;49(7):e13118.
19. Chambless LE, Heiss G, Folsom AR, Rosamond W, Szklo M, Sharrett AR, et al. Association of coronary heart disease incidence with carotid arterial wall thickness and major risk factors: the Atherosclerosis Risk in Communities (ARIC) Study, 1987-1993. Am J Epidemiol. 1997;146(6):483-94.
20. Chambless LE, Folsom AR, Clegg LX, Sharrett AR, Shahar E, Nieto FJ, et al. Carotid wall thickness is predictive of incident clinical stroke: the Atherosclerosis Risk in Communities (ARIC) study. Am J Epidemiol. 2000;151(5):478-87.
21. den Hoed M, Strawbridge RJ, Almgren P, Gustafsson S, Axelsson T, Engstrom G, et al. GWAS-identified loci for coronary heart disease are associated with intima-media thickness and plaque presence at the carotid artery bulb. Atherosclerosis. 2015;239(2):304-10.
22. Magnusson M, Lewis GD, Ericson U, Orho-Melander M, Hedblad B, Engstrom G, et al. A diabetes-predictive amino acid score and future cardiovascular disease. Eur Heart J. 2013;34(26):1982-9.
23. Baldassarre D, Hamsten A, Veglia F, de Faire U, Humphries SE, Smit AJ, et al. Measurements of carotid intima-media thickness and of interadventitia common carotid diameter improve prediction of cardiovascular events: results of the IMPROVE (Carotid Intima Media Thickness [IMT] and IMT-Progression as Predictors of Vascular Events in a High Risk European Population) study. J Am Coll Cardiol. 2012;60(16):1489-99.
24. Andersson J, Sundstrom J, Gustavsson T, Hulthe J, Elmgren A, Zilmer K, et al. Echogenecity of the carotid intima-media complex is related to cardiovascular risk factors, dyslipidemia, oxidative stress and inflammation: the Prospective Investigation of the Vasculature in Uppsala Seniors (PIVUS) study. Atherosclerosis. 2009;204(2):612-8.
25. Epidemiology of Diabetes I, Complications Research G. Effect of intensive diabetes treatment on carotid artery wall thickness in the epidemiology of diabetes interventions and complications. Epidemiology of Diabetes Interventions and Complications (EDIC) Research Group. Diabetes. 1999;48(2):383-90.
26. Baldassarre D, Amato M, Pustina L, Tremoli E, Sirtori CR, Calabresi L, et al. Increased carotid artery intima-media thickness in subjects with primary hypoalphalipoproteinemia. Arterioscler Thromb Vasc Biol. 2002;22(2):317-22.
27. Karvonen J, Kauma H, Kervinen K, Rantala M, Ikaheimo M, Paivansalo M, et al. Endothelial nitric oxide synthase gene Glu298Asp polymorphism and blood pressure, left ventricular mass and carotid artery atherosclerosis in a population-based cohort. J Intern Med. 2002;251(2):102-10.
28. Gaenzer H, Marschang P, Sturm W, Neumayr G, Vogel W, Patsch J, et al. Association between increased iron stores and impaired endothelial function in patients with hereditary hemochromatosis. J Am Coll Cardiol. 2002;40(12):2189-94.
29. Strohmer B, Pichler M, Iglseder B, Paulweber B. Relationship of QT interval duration with carotid intima media thickness in a clinically healthy population undergoing cardiovascular risk screening. J Intern Med. 2005;257(3):238-46.
30. Astrand H, Ryden-Ahlgren A, Sandgren T, Lanne T. Age-related increase in wall stress of the human abdominal aorta: an in vivo study. J Vasc Surg. 2005;42(5):926-31.
31. de Vries R, Perton FG, Dallinga-Thie GM, van Roon AM, Wolffenbuttel BH, van Tol A, et al. Plasma cholesteryl ester transfer is a determinant of intima-media thickness in type 2 diabetic and nondiabetic subjects: role of CETP and triglycerides. Diabetes. 2005;54(12):3554-9.
32. Poykko SM, Kellokoski E, Ukkola O, Kauma H, Paivansalo M, Kesaniemi YA, et al. Plasma ghrelin concentrations are positively associated with carotid artery atherosclerosis in males. J Intern Med. 2006;260(1):43-52.
33. Cardellini M, Marini MA, Frontoni S, Hribal ML, Andreozzi F, Perticone F, et al. Carotid artery intima-media thickness is associated with insulin-mediated glucose disposal in nondiabetic normotensive offspring of type 2 diabetic patients. Am J Physiol Endocrinol Metab. 2007;292(1):E347-52.
34. Fan AZ, Paul-Labrador M, Merz CN, Iribarren C, Dwyer JH. Smoking status and common carotid artery intima-medial thickness among middle-aged men and women based on ultrasound measurement: a cohort study. BMC Cardiovasc Disord. 2006;6:42.

35. Kawamoto R, Tomita H, Inoue A, Ohtsuka N, Kamitani A. Metabolic syndrome may be a risk factor for early carotid atherosclerosis in women but not in men. J Atheroscler Thromb. 2007;14(1):36-43.
36. Pertovaara M, Raitala A, Juonala M, Lehtimaki T, Huhtala H, Oja SS, et al. Indoleamine 2,3-dioxygenase enzyme activity correlates with risk factors for atherosclerosis: the Cardiovascular Risk in Young Finns Study. Clin Exp Immunol. 2007;148(1):106-11.
37. Chang HS, Kim HC, Ahn SV, Hur NW, Suh I. Impact of multiple cardiovascular risk factors on the carotid intima-media thickness in young adults: the Kangwha Study. J Prev Med Public Health. 2007;40(5):411-7.
38. Lee YJ, Nam CM, Kim HC, Hur NW, Suh I. [The association between obesity indices in adolescence and carotid intima-media thickness in young adults: Kangwha Study]. J Prev Med Public Health. 2008;41(2):107-14.
39. O'Donnell CJ, Demissie S, Kimura M, Levy D, Gardner JP, White C, et al. Leukocyte telomere length and carotid artery intimal medial thickness: the Framingham Heart Study. Arterioscler Thromb Vasc Biol. 2008;28(6):1165-71.
40. Romero JR, Vasan RS, Beiser AS, Polak JF, Benjamin EJ, Wolf PA, et al. Association of carotid artery atherosclerosis with circulating biomarkers of extracellular matrix remodeling: the Framingham Offspring Study. J Stroke Cerebrovasc Dis. 2008;17(6):412-7.
41. Bertoni AG, Whitt-Glover MC, Chung H, Le KY, Barr RG, Mahesh M, et al. The association between physical activity and subclinical atherosclerosis: the Multi-Ethnic Study of Atherosclerosis. Am J Epidemiol. 2009;169(4):444-54.
42. Jylhava J, Eklund C, Pessi T, Raitakari OT, Juonala M, Kahonen M, et al. Genetics of C-reactive protein and complement factor H have an epistatic effect on carotid artery compliance: the Cardiovascular Risk in Young Finns Study. Clin Exp Immunol. 2009;155(1):53-8.
43. Paramo JA, Beloqui O, Rodriguez JA, Diez J, Orbe J. Association between matrix metalloproteinase-10 concentration and smoking in individuals without cardiovascular disease. Rev Esp Cardiol. 2008;61(12):1267-73.
44. Suh M, Lee JY, Ahn SV, Kim HC, Suh I. [C-reactive protein and carotid intima-media thickness in a population of middle-aged Koreans]. J Prev Med Public Health. 2009;42(1):29-34.
45. Jylhava J, Haarala A, Eklund C, Pertovaara M, Kahonen M, Hutri-Kahonen N, et al. Serum amyloid A is independently associated with metabolic risk factors but not with early atherosclerosis: the Cardiovascular Risk in Young Finns Study. J Intern Med. 2009;266(3):286-95.
46. Shah AS, Dolan LM, Kimball TR, Gao Z, Khoury PR, Daniels SR, et al. Influence of duration of diabetes, glycemic control, and traditional cardiovascular risk factors on early atherosclerotic vascular changes in adolescents and young adults with type 2 diabetes mellitus. J Clin Endocrinol Metab. 2009;94(10):3740-5.
47. Hyder JA, Allison MA, Barrett-Connor E, Detrano R, Wong ND, Sirlin C, et al. Bone mineral density and atherosclerosis: the Multi-Ethnic Study of Atherosclerosis, Abdominal Aortic Calcium Study. Atherosclerosis. 2010;209(1):283-9.
48. Sojkova J, Najjar SS, Beason-Held LL, Metter EJ, Davatzikos C, Kraut MA, et al. Intima-media thickness and regional cerebral blood flow in older adults. Stroke. 2010;41(2):273-9.
49. Lee YH, Shin MH, Kweon SS, Rhee JA, Ryu SY, Ahn HR, et al. Metabolic syndrome and carotid artery parameter in Koreans aged 50 years and older. Circ J. 2010;74(3):560-6.
50. Uurtuya S, Kotani K, Taniguchi N, Yoshioka H, Kario K, Ishibashi S, et al. Comparative study of atherosclerotic parameters in Mongolian and Japanese patients with hypertension and diabetes mellitus. J Atheroscler Thromb. 2010;17(2):181-8.
51. Liao YC, Lin HF, Guo YC, Yu ML, Liu CK, Juo SH. Sex-differential genetic effect of phosphodiesterase 4D (PDE4D) on carotid atherosclerosis. BMC Med Genet. 2010;11:93.
52. Chumaeva N, Hintsanen M, Juonala M, Raitakari OT, Keltikangas-Jarvinen L. Sex differences in the combined effect of chronic stress with impaired vascular endothelium functioning and the development of early atherosclerosis: the Cardiovascular Risk in Young Finns study. BMC Cardiovasc Disord. 2010;10:34.
53. Lin HF, Tsai PC, Lin RT, Khor GT, Sheu SH, Juo SH. Sex differential genetic effect of chromosome 9p21 on subclinical atherosclerosis. PLoS One. 2010;5(11):e15124.

54. Irace C, Carallo C, De Franceschi MS, Scicchitano F, Milano M, Tripolino C, et al. Human common carotid wall shear stress as a function of age and gender: a 12-year follow-up study. Age (Dordr). 2012;34(6):1553-62.
55. Franck N, Lanne T, Astrand O, Engvall J, Lindstrom T, Ostgren CJ, et al. Cardiovascular risk factors related to the PPARgamma Pro12Ala polymorphism in patients with type 2 diabetes are gender dependent. Blood Press. 2012;21(2):122-7.
56. Rossi IA, Bochud M, Viswanathan B, Riesen W, Bovet P. Relation between high-sensitivity C-reactive protein and cardiovascular and renal markers in a middle-income country in the African region. Int J Cardiol. 2012;156(2):203-8.
57. Janicki-Deverts D, Cohen S, Matthews KA, Jacobs DR, Jr., Adler NE. Occupational mobility and carotid artery intima-media thickness: findings from the Coronary Artery Risk Development in Young Adults Study. Psychosom Med. 2011;73(9):795-802.
58. Gomez-Marcos MA, Recio-Rodriguez JI, Patino-Alonso MC, Agudo-Conde C, Gomez-Sanchez L, Rodriguez-Sanchez E, et al. Relationships between high-sensitive C-reactive protein and markers of arterial stiffness in hypertensive patients. Differences by sex. BMC Cardiovasc Disord. 2012;12:37.
59. Herder M, Johnsen SH, Arntzen KA, Mathiesen EB. Risk factors for progression of carotid intima-media thickness and total plaque area: a 13-year follow-up study: the Tromso Study. Stroke. 2012;43(7):1818-23.
60. Cipolli JA, Ferreira-Sae MC, Martins RP, Pio-Magalhaes JA, Bellinazzi VR, Matos-Souza JR, et al. Relationship between serum uric acid and internal carotid resistive index in hypertensive women: a cross-sectional study. BMC Cardiovasc Disord. 2012;12:52.
61. Su TC, Chien KL, Jeng JS, Chen MF, Hsu HC, Torng PL, et al. Age- and gender-associated determinants of carotid intima-media thickness: a community-based study. J Atheroscler Thromb. 2012;19(9):872-80.
62. Dahlen EM, Bjarnegard N, Lanne T, Nystrom FH, Ostgren CJ. Sagittal abdominal diameter is a more independent measure compared with waist circumference to predict arterial stiffness in subjects with type 2 diabetes--a prospective observational cohort study. Cardiovasc Diabetol. 2013;12:55.
63. Itkonen ST, Karp HJ, Kemi VE, Kokkonen EM, Saarnio EM, Pekkinen MH, et al. Associations among total and food additive phosphorus intake and carotid intima-media thickness--a cross-sectional study in a middle-aged population in Southern Finland. Nutr J. 2013;12:94.
64. Kim J, Pack A, Maislin G, Lee SK, Kim SH, Shin C. Prospective observation on the association of snoring with subclinical changes in carotid atherosclerosis over four years. Sleep Med. 2014;15(7):769-75.
65. Spartano NL, Augustine JA, Lefferts WK, Gump BB, Heffernan KS. The relationship between carotid blood pressure reactivity to mental stress and carotid intima-media thickness. Atherosclerosis. 2014;236(2):227-9.
66. Kedenko L, Lamina C, Kedenko I, Kollerits B, Kiesslich T, Iglseder B, et al. Genetic polymorphisms at SIRT1 and FOXO1 are associated with carotid atherosclerosis in the SAPHIR cohort. BMC Med Genet. 2014;15:112.
67. Lee YH, Shin MH, Choi JS, Nam HS, Jeong SK, Park KS, et al. Gender differences in the association between depressive symptoms and carotid atherosclerosis among middle-aged and older Koreans: the Namwon study. J Korean Med Sci. 2014;29(11):1507-13.
68. Rosvall M, Persson M, Ostling G, Nilsson PM, Melander O, Hedblad B, et al. Risk factors for the progression of carotid intima-media thickness over a 16-year follow-up period: the Malmo Diet and Cancer Study. Atherosclerosis. 2015;239(2):615-21.
69. Jones MR, Diez-Roux AV, O'Neill MS, Guallar E, Sharrett AR, Post W, et al. Ambient air pollution and racial/ethnic differences in carotid intima-media thickness in the Multi-Ethnic Study of Atherosclerosis (MESA). J Epidemiol Community Health. 2015;69(12):1191-8.
70. Persson J, Strawbridge RJ, McLeod O, Gertow K, Silveira A, Baldassarre D, et al. Sex-Specific Effects of Adiponectin on Carotid Intima-Media Thickness and Incident Cardiovascular Disease. J Am Heart Assoc. 2015;4(8):e001853.
71. Ceponiene I, Klumbiene J, Tamuleviciute-Prasciene E, Motiejunaite J, Sakyte E, Ceponis J, et al. Associations between risk factors in childhood (12-13 years) and adulthood (48-49 years) and subclinical atherosclerosis: the Kaunas Cardiovascular Risk Cohort Study. BMC Cardiovasc Disord. 2015;15:89.

72. Gomez-Marcos MA, Recio-Rodriguez JI, Gomez-Sanchez L, Agudo-Conde C, Rodriguez-Sanchez E, Maderuelo-Fernandez J, et al. Gender differences in the progression of target organ damage in patients with increased insulin resistance: the LOD-DIABETES study. Cardiovasc Diabetol. 2015;14:132.
73. Shen H, Xu L, Lu J, Hao T, Ma C, Yang H, et al. Correlation between small dense low-density lipoprotein cholesterol and carotid artery intima-media thickness in a healthy Chinese population. Lipids Health Dis. 2015;14:137.
74. Hakulinen C, Pulkki-Raback L, Elovainio M, Kubzansky LD, Jokela M, Hintsanen M, et al. Childhood Psychosocial Cumulative Risks and Carotid Intima-Media Thickness in Adulthood: The Cardiovascular Risk in Young Finns Study. Psychosom Med. 2016;78(2):171-81.
75. Shin J, Park JH, Song YM, Lee K, Sung J. Association between Lumbar Bone Mineral Density and Carotid Intima-Media Thickness in Korean Adults: a Cross-sectional Study of Healthy Twin Study. J Korean Med Sci. 2017;32(1):70-6.
76. Thorsson B, Eiriksdottir G, Sigurdsson S, Gudmundsson EF, Bots ML, Aspelund T, et al. Population distribution of traditional and the emerging cardiovascular risk factors carotid plaque and IMT: the REFINE-Reykjavik study with comparison with the Tromso study. BMJ Open. 2018;8(5):e019385.
77. Sigurdsson E, Thorgeirsson G, Sigvaldason H, Sigfusson N. Prevalence of coronary heart disease in Icelandic men 1968-1986. The Reykjavik Study. Eur Heart J. 1993;14(5):584-91.
78. Yasuda M, Sato H, Hashimoto K, Osada U, Hariya T, Nakayama H, et al. Carotid artery intima-media thickness, HDL cholesterol levels, and gender associated with poor visual acuity in patients with branch retinal artery occlusion. PLoS One. 2020;15(10):e0240977.
79. Holwerda SW, Luehrs RE, DuBose LE, Majee R, Pierce GL. Sex and age differences in the association between sympathetic outflow and central elastic artery wall thickness in humans. Am J Physiol Heart Circ Physiol. 2019;317(3):H552-H60.
80. Gomez-Marcos MA, Gomez-Sanchez L, Patino-Alonso MC, Recio-Rodriguez JI, Gomez-Sanchez M, Rigo F, et al. A body shape index and vascular structure and function in Spanish adults (MARK study): A cross-sectional study. Medicine (Baltimore). 2018;97(47):e13299.
81. Hanna DB, Moon JY, Haberlen SA, French AL, Palella FJ, Jr., Gange SJ, et al. Carotid artery atherosclerosis is associated with mortality in HIV-positive women and men. AIDS. 2018;32(16):2393-403.
82. Basu A, Jenkins AJ, Stoner JA, Zhang Y, Klein RL, Lopes-Virella MF, et al. Apolipoprotein-defined lipoprotein subclasses, serum apolipoproteins, and carotid intima-media thickness in T1D. J Lipid Res. 2018;59(5):872-83.
83. Sturlaugsdottir R, Aspelund T, Bjornsdottir G, Sigurdsson S, Thorsson B, Eiriksdottir G, et al. Prevalence and determinants of carotid plaque in the cross-sectional REFINE-Reykjavik study. BMJ Open. 2016;6(11):e012457.
84. Ma H, Lin H, Hu Y, Li X, He W, Jin X, et al. Relationship between non-high-density lipoprotein cholesterol and carotid atherosclerosis in normotensive and euglycemic Chinese middle-aged and elderly adults. Lipids Health Dis. 2017;16(1):55.
85. Rosfors S, Hallerstam S, Jensen-Urstad K, Zetterling M, Carlstrom C. Relationship between intima-media thickness in the common carotid artery and atherosclerosis in the carotid bifurcation. Stroke. 1998;29(7):1378-82.
86. Kapiotis S, Holzer G, Schaller G, Haumer M, Widhalm H, Weghuber D, et al. A proinflammatory state is detectable in obese children and is accompanied by functional and morphological vascular changes. Arterioscler Thromb Vasc Biol. 2006;26(11):2541-6.
87. Nyasordzi J, Penczynski K, Remer T, Buyken AE. Early life factors and their relevance to intima-media thickness of the common carotid artery in early adulthood. PLoS One. 2020;15(5):e0233227.
88. Maloberti A, Qualliu E, Occhi L, Sun J, Grasso E, Tognola C, et al. Hyperuricemia prevalence in healthy subjects and its relationship with cardiovascular target organ damage. Nutr Metab Cardiovasc Dis. 2021;31(1):178-85.
89. Karikkineth AC, AlGhatrif M, Oberdier MT, Morrell C, Palchamy E, Strait JB, et al. Sex Differences in Longitudinal Determinants of Carotid Intima Medial Thickening With Aging in a Community-Dwelling Population: The Baltimore Longitudinal Study on Aging. J Am Heart Assoc. 2020;9(22):e015396.
90. Wang Z, Li W, Liu W, Tian J. Gender is a determinant of carotid artery stiffness independent of age and blood pressure. Br J Radiol. 2021;94(1119):20200796.
